# The essential role of sugar metabolism in the acclimation response of *Arabidopsis thaliana* to high light intensities

**DOI:** 10.1093/jxb/eru027

**Published:** 2014-02-12

**Authors:** Jessica Schmitz, Luisa Heinrichs, Federico Scossa, Alisdair R. Fernie, Marie-Luise Oelze, Karl-Josef Dietz, Maxi Rothbart, Bernhard Grimm, Ulf-Ingo Flügge, Rainer E. Häusler

**Affiliations:** ^1^Department of Botany II, Cologne Biocenter, University of Cologne, Zülpicher Straße 47b, D-50674 Cologne, Germany; ^2^Max-Planck-Institute of Molecular Plant Physiology, Am Mühlenberg 1, D-14476 Potsdam OT Golm, Germany; ^3^Agriculture Research Council, Research Center for Vegetable Crops, Via Cavalleggeri 25, 84098 Pontecagnano (Salerno), Italy; ^4^Biochemistry and Physiology of Plants, University of Bielefeld, Universitätsstraße 25, D-33615 Bielefeld, Germany; ^5^Institute of Biology, Plant Physiology, Humboldt-University Berlin, Philippstraße 13, D-10115 Berlin, Germany

**Keywords:** *Arabidopsis* mutants, carbon partitioning, light acclimation, metabolomics, photosynthesis, retrograde signalling, sugar metabolism, transcriptomics.

## Abstract

Retrograde signals from chloroplasts are thought to control the expression of nuclear genes associated with plastidial processes such as acclimation to varying light conditions. *Arabidopsis* mutants altered in the day and night path of photoassimilate export from the chloroplasts served as tools to study the involvement of carbohydrates in high light (HL) acclimation. A double mutant impaired in the triose phosphate/phosphate translocator (TPT) and ADP-glucose pyrophosphorylase (AGPase) (*adg1-1/tpt-2*) exhibits a HL-dependent depletion in endogenous carbohydrates combined with a severe growth and photosynthesis phenotype. The acclimation response of mutant and wild-type plants has been assessed in time series after transfer from low light (LL) to HL by analysing photosynthetic performance, carbohydrates, MgProtoIX (a chlorophyll precursor), and the ascorbate/glutathione redox system, combined with microarray-based transcriptomic and GC-MS-based metabolomic approaches. The data indicate that the accumulation of soluble carbohydrates (predominantly glucose) acts as a short-term response to HL exposure in both mutant and wild-type plants. Only if carbohydrates are depleted in the long term (e.g. after 2 d) is the acclimation response impaired, as observed in the *adg1-1/tpt-2* double mutant. Furthermore, meta-analyses conducted with in-house and publicly available microarray data suggest that, in the long term, reactive oxygen species such as H_2_O_2_ can replace carbohydrates as signals. Moreover, a cross-talk exists between genes associated with the regulation of starch and lipid metabolism. The involvement of genes responding to phytohormones in HL acclimation appears to be less likely. Various candidate genes involved in retrograde control of nuclear gene expression emerged from the analyses of global gene expression.

## Summary

Analyses of mutants impaired in assimilate export from chloroplasts revealed that carbohydrates as primary output of photosynthesis control expression of nuclear genes associated with plastidial processes such as acclimation to high light intensities.

## Introduction

Light is one of the most prominent environmental factors for plants. It not only determines the rate of photosynthesis and biomass production (Sharkey, 1985), but it also controls various aspects of development (Frankhauser and Chory, 1997). Apart from these beneficial effects, excess light can trigger metabolic changes as well as redox imbalances, and thus has the potential to damage thylakoid structures in the chloroplasts. Therefore, the activity and composition of thylakoid proteins, which determine photosynthetic electron transport rates (ETRs), have to be adapted to changing light intensities over both short- and long-term periods. Long-term adaptation can be ascribed as acclimation (Eberhard *et al.*, 2008; Kleine *et al.*, 2009).

The vast majority of chloroplast-localized proteins are nuclear encoded (Leister, 2003). In order to adjust their expression to plastome-encoded photosynthesis genes, a constant flow of information from the chloroplast to the nucleus seems to be required (Beck, 2005; Koussevitzky *et al.*, 2007; Eberhard *et al.*, 2008). It has been proposed that this ‘retrograde signalling’ is based on a variety of components such as tetrapyrroles (Surpin *et al.*, 2002; Beck, 2005; Czarnecki *et al.*, 2012), reactive oxygen species (ROS; Kim *et al.*, 2008; Foyer and Noctor, 2009; Miller *et al.*, 2009; Triantaphylidès and Havaux, 2009; Balazadeh *et al.*, 2012), the redox state of plastoquinone (Pfannschmidt *et al.*, 1999; Pfalz *et al.*, 2012), plastidial gene expression (Ahlert *et al.*, 2003), the metabolic state of the mesophyll including sugars (Smeekens, 1998, 2000; Rolland *et al.*, 2002, 2006; Bräutigam *et al.*, 2009), and plant hormones, such as abscisic acid (ABA), as a response to an enhanced xanthophyll cycle activity (Baier *et al.*, 2004; Baier and Dietz, 2005). More recently phosphoadenosine phosphate (PAP; Estavillo *et al.*, 2012) and 2-*C*-methyl-d-erythritol-2,4-cyclopyrophosphate (MecPP; Xiao *et al.*, 2012), an intermediate of the plastidial methylerythritol phosphate (MEP) pathway of isoprenoid biosynthesis (Lichtenthaler, 1999), have also been proposed to act as retrograde signals. Despite a long list of compounds and/or conditions involved, detailed information on how these retrograde signals are integrated and modify nuclear gene expression is still missing. It is also conceivable that retrograde signalling is less complex than believed and relies only on a limited number of primary signals, which then use known components, for example those involved in sugar or hormonal signalling (for a review, see Rolland *et al.*, 2006). Mutants defective in the day and night path of photoassimilate export from the chloroplasts can serve as a model system to test this assumption.

The export of triose phosphates (TPs) during the day (day path) is blocked by a knockout of the TP/phosphate translocator (TPT; Schneider *et al.*, 2002). The absence of a functional ADP-glucose pyrophosphorylase (AGPase) in the *adg1-1* mutant in turn results in the absence of starch (Lin *et al*., 1986) and therefore a block in maltose and glucose delivery and export during the night (night path). In contrast, the *adg1-1* mutant accumulates high amounts of soluble sugars during the day (Kunz *et al.*, 2010; Schmitz *et al.*, 2012). While a defect in the TPT can be compensated for by an increased starch turnover in the light (Häusler *et al.*, 1998; Schneider *et al.*, 2002; Walters *et al.*, 2004), a simultaneous block in TPT and AGPase in the *adg1-1/tpt-2* double mutant results in severe growth retardation, high chlorophyll fluorescence, inhibition of the ETR, and depletion of carbohydrates (Häusler *et al.*, 2009; Schmitz *et al.*, 2012). This harsh phenotype conditionally occurs only under high light (HL) conditions, but is absent in low light (LL)-grown plants (Schmitz *et al.*, 2012). Marked differences in the abundance of plastome- and nuclear-encoded thylakoid proteins in *adg1-1/tpt-2* were also HL dependent and could be rescued by feeding sugars to the plants (Heinrichs *et al.*, 2012; Schmitz *et al.*, 2012), suggesting that (i) HL acclimation is impaired in the double mutant and (ii) soluble sugars can restore the acclimation response.

The response of wild-type and mutant plants to continuous growth in HL or LL has been extensively characterized by Schmitz *et al.* (2012). Here, time series rather than different light intensities (Walters *et al.*, 2004) have been used to study the impact of impaired carbon partitioning and leaf carbohydrate metabolism on the acclimation response of *Arabidopsis thaliana* towards HL. Photosynthesis parameters, metabolite levels, and global gene expression were measured after LL to HL (LL/HL) transfer in mutant and wild-type plants. The data suggest that endogenous sugars and ROS play a pivotal role in HL acclimation in *A. thaliana*.

## Materials and methods

### Plant material, growth conditions, and photosynthesis measurements

Plant material and growth conditions in HL [photon flux density (PFD=300 μmol m^–2^ s^–1^) or LL (PFD=30 μmol m^–2^ s^–1^) have been described in detail by Schmitz *et al.* (2012). For LL/HL transfer experiments, plants were grown under long-day conditions (i.e. 16h light/8h dark) for 3 weeks. Photosynthesis parameters at photosystem II (PSII) were determined according to Schreiber *et al*. (1986) and Genty *et al.* (1989) with an IMAGING-PAM fluorometer (Walz, Effeltrich, Germany).

### Determination of metabolites

Carbohydrates were extracted and measured enzymatically as described by Schmitz *et al.* (2012). Anthocyanins were determined according to Giraud *et al.* (2008).

For gas chromatography–time of flight-mass spectrometry (GC-ToF-MS) profiling, primary metabolites were extracted and derivatized according to established procedures (Lisec *et al.*, 2006). Raw data were then normalized following the procedure outlined in Roessner *et al.* (2001); peaks were identified using the software Tagfinder (Luedemann *et al.*, 2012), with the support of the mass tags deposited in the Golm Metabolome Database (Kopka *et al.*, 2005). Further details regarding the metabolic profiling are reported in Supplementary Table S1 available at *JXB* online, which is compliant with the recommendations reported in Fernie *et al.* (2011).

Ascorbic acid (Asc) and dehydroascorbate (DHA) were quantified with ascorbate oxidase according to Horling *et al.* (2003), and glutathione (GSH) and glutathione disulphide (GSSG) by enzymatic cycling with glutathione reductase and 5,5′-dithiobis (2-nitrobenzoic acid) (DTNB) (Griffith, 1980) with some modifications, as described by Oelze *et al.* (2012).

Mg-protoporphrin IX (MgProtoIX) was extracted from 100mg of frozen leaf powder in 2×200 μl and 1×100 μl acetone:methanol:0.1 N NH_4_OH (10:9:1). After each centrifugation for 10min at 4 °C, the supernatants were collected and aliquots analysed by HPLC-based fluorescence detection to quantify Mg-porphyrins by means of authentic standards (Frontier Scientific) according to Czarnecki *et al.* (2011).

### RNA extraction for transcriptome analyses and quantitative reverse transcription–PCR (qRT–PCR)

Samples for transcriptome analyses were collected from two sets of independently grown plants, with 3–6 plants per sample. Total RNA was isolated according to Logemann *et al.* (1987), and for transcriptome analyses subsequently purified on Plant RNeasy extraction columns (Qiagen). Processing and hybridization to GeneChip ATH1 Genome Arrays (Affymetrix) was conducted at the ‘Kompetenzzentrum Fluoreszente Bioanalytik’ (KFB, Regensburg). The complete set of microarray data can be obtained from http://www.ebi.ac.uk/miamexpress/ (ArrayExpress accession: E-MEXP-3791). For qRT–PCR with *Lhcb1* the following primer combination was used (RL_LhcbI_fwd: TTCCCTGGAGACTACGGATG; RL_LhcbI_rev: CCCACCTGCTGTGGATAACT).

### Statistical evaluation of experimental data

Raw fluorescence probe signals of Affymetrix microarrays were evaluated and statistically analysed with the Robin software (Lohse *et al.*, 2010). The data were normalized by the RMA-method (robust multiarray averaging; Wu *et al.*, 2004). Average log2-fold expression changes and adjusted *P*-values were calculated by Linear Models for Microarray Data (Limma; Smyth, 2004) and false discovery rate (FDR) corrected according to Benjamini and Hochberg (1995). The Tair10 genome release as well as a modified Mapman Binning (Thimm *et al.*, 2004) were used to annotate the probe-IDs. Venn diagrams were constructed with the help of the web-based tool Venny (Oliveros, 2007). The R-tool 2.12.0 (package ‘pcaMethods’; Stacklies *et al.*, 2007) has been implemented for multivariate analyses [principle component analysis (PCA)] as well as for Fisher’s exact tests.

Significant differences between more than two physiological data sets were analysed using single-site analysis of variance (ANOVA) combined with the post-hoc Tukey–Kramer test, which allows the comparison of unequal sample sizes and identifies values which are significantly different (Ludbrook, 1998). For data plotting and fitting, SigmaPlot10.0 for Windows (SPSS Inc.) was used.

Published microarray data were obtained either from NASC (http://affymetrix.arabidopsis.info) or from the supplementary data of the respective publications online. Differentially regulated genes were filtered by log2-fold changes at a threshold of ±1 (for time series at least one time point had to contain the candidate gene at a threshold of ±1) and commonly regulated genes were extracted. The relative abundance of coinciding genes was calculated from $A_{c}=\;\sqrt{\frac{a_{0}}{X_{T}}•\frac{a_{0}}{Y_{T}}}$, where A_c_ is the coincidence coefficient, and a_0_ equals the number of coinciding genes as a fraction of the total number of differentially regulated genes in in-house (X_T_) or published (Y_T_) experiments. The sum of the A_c_ values of all foreign arrays was set to 100% and, from the relative contribution, pie charts were constructed. The correlation of log2 ratios between in-house and published arrays was calculated according to Pearson (Galton, 1888).

## Results and Discussion

### LL/HL transfer leads to rapid photoinhibition followed by a slow recovery

**Fig. 1.**
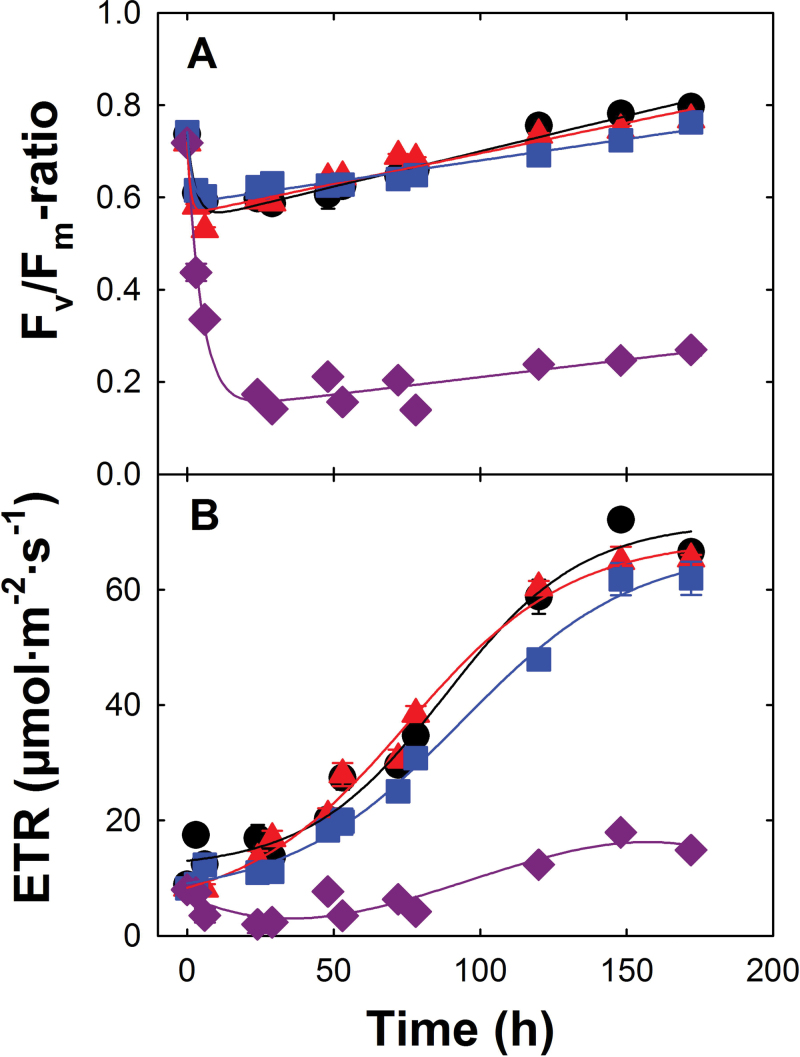
Time-dependent response of Chl *a* fluorescence in the dark-adapted state and photosynthetic ETR upon LL/HL transfer of wild-type and mutant plants. Col-0 (circles), *tpt-2* (squares), *adg1-1* (triangles), and *adg1-1/tpt-2* (diamonds) plants were grown for 3 weeks under LL and long-day conditions. The plants were transferred to HL at *t*=0 (4h in the light). The maximum quantum yield of ETR through PSII is reflected by the *F*_v_/*F*_m_ ratio (A). The ETR (B) was determined at a PFD of ~300 μmol m^–2^ s^–1^. The data represent the mean ±SE of *n*=3 replicates. In some cases, the error bars are smaller than the symbols. (This figure is available in colour at *JXB* online.)

Exposure of LL-grown plants to HL conditions rapidly leads to severe photoinhibition and a re-organization of the photosynthetic apparatus during acclimation (Bailey *et al.*, 2004; Horton *et al.*, 2008). The dynamics of these processes are reflected in changes of the photosynthetic light reaction determined by PAM fluorometry. Modulated chlorophyll *a* (Chl *a*) fluorescence in dark-adapted mutant and wild-type plants was monitored over a time period of 1 week (172h) after LL/HL transfer (Fig. 1). HL exposure of LL-grown plants resulted in a drop in the *F*_v_/*F*_m_ ratio within the first 3h based on photoinhibition, followed by a slow recovery (Fig. 1A). In contrast to wild-type and single mutant plants, which exhibit a complete recovery of *F*_v_/*F*_m_ within 1 week, the *F*_v_/*F*_m_ ratio in *adg1-1/tpt-2* decreased to a minimum value below 0.2 within the first 2 d upon HL exposure, followed by a small, but incomplete recovery thereafter. The decrease in *F*_v_/*F*_m_ can be attributed to the dissociation of light-harvesting complexes (LHCs) from photosynthetic core complexes (Schmitz *et al.*, 2012). The ETR in all plant lines increased only slowly with time after LL/HL transfer. In Col-0, *tpt-2*, and *adg1-1*, the maximum increase in ETR commenced after 48h in HL (Fig. 1B), whereas in the *adg1-1/tpt-2* double mutant the ETR remained significantly lower over the whole time course of the experiment. The statistical analysis of the above data is contained in Table 1 of Supplementary Document S1 available at *JXB* online.

**Table 1.** Distribution of functional categories following the ‘static’ assessment of expression profiles of *adg1-1, tpt-2*, and *adg1-1/tpt-2* versus wild-type plants at *t*_0_ (LL) and at *t*_4h_ and *t*_48h_ after LL/HL transferOnly functional categories with five or more altered genes are displayed.

Functional categories	*adg1-1* versus Col-0						*tpt-2* versus Col-0		*adg1-1/tpt-2* versus Col-0	
	*t*_0_		*t*_4h_		*t*_48h_		*t*_4h_	*t*_48h_	*t*_48h_	
	Up	Down	Up	Down	Up	Down	Up	Up	Up	Down
Amino acid metabolism	–	–	–	–	–	–	–	–	–	5 (4.4)*
Cell wall	–	–	–	–	–	–	–	6 (2.7)*	–	11 (4.6)*
CHO metabolism	–	–	–	–	5 (8.7)*	–	–	–	–	6 (5.4)*
Development	5 (2.4)	–	–	–	–	–	–	–	–	–
Hormone metabolism	–	–	–	–	–	–	–	6 (2.8)*	–	–
Lipid metabolism	–	–	5 (10.2)*	–	–	7 (2.8)*	–	–	–	–
Protein – degradation	–	11 (2.3)*	–	–	–	**14 (1.4)**	6 (2.6)*	5 (0.7)	–	–
Protein – synthesis	–	–	–	–	7 (5.2)*	–	–	–	–	–
Protein – synthesis (plastome)	–	–	–	–	–	–	–	–	5 (140)*	–
PS – light reaction (plastome)	–	–	–	–	–	–	–	–	16 (129)*	–
RNA – regulation of transcription	–	–	–	–	6 (1.2)	21 (1.6)	–	6 (0.7)	–	9 (0.9)
Signalling	–	–	–	–	–	7 (0.9)	–	10 (1.7)	–	–
Stress	–	–	–	5 (8.1)*	–	11 (2.0)*	–	12 (3.2)*	–	–
Transport	–	–	–	–	–	5 (0.8)	–	8 (1.9)	–	5 (1.1)

The numbers of significantly (*P*<0.01) altered genes were taken from Supplementary Tables S2–S4 available at *JXB* online.

The threshold of log2 ratios was kept at ±1.0.

The data in parentheses refer to over- or under-represented functional categories (i.e. numbers above or below 1).

Significantly (*P*<0.05) over- or under-represented categories according to Fisher’s exact test are marked by an asterisk.

Over-represented categories are shown in bold.

### LL/HL transfer results in sugar and anthocyanin accumulation

**Fig. 2.**
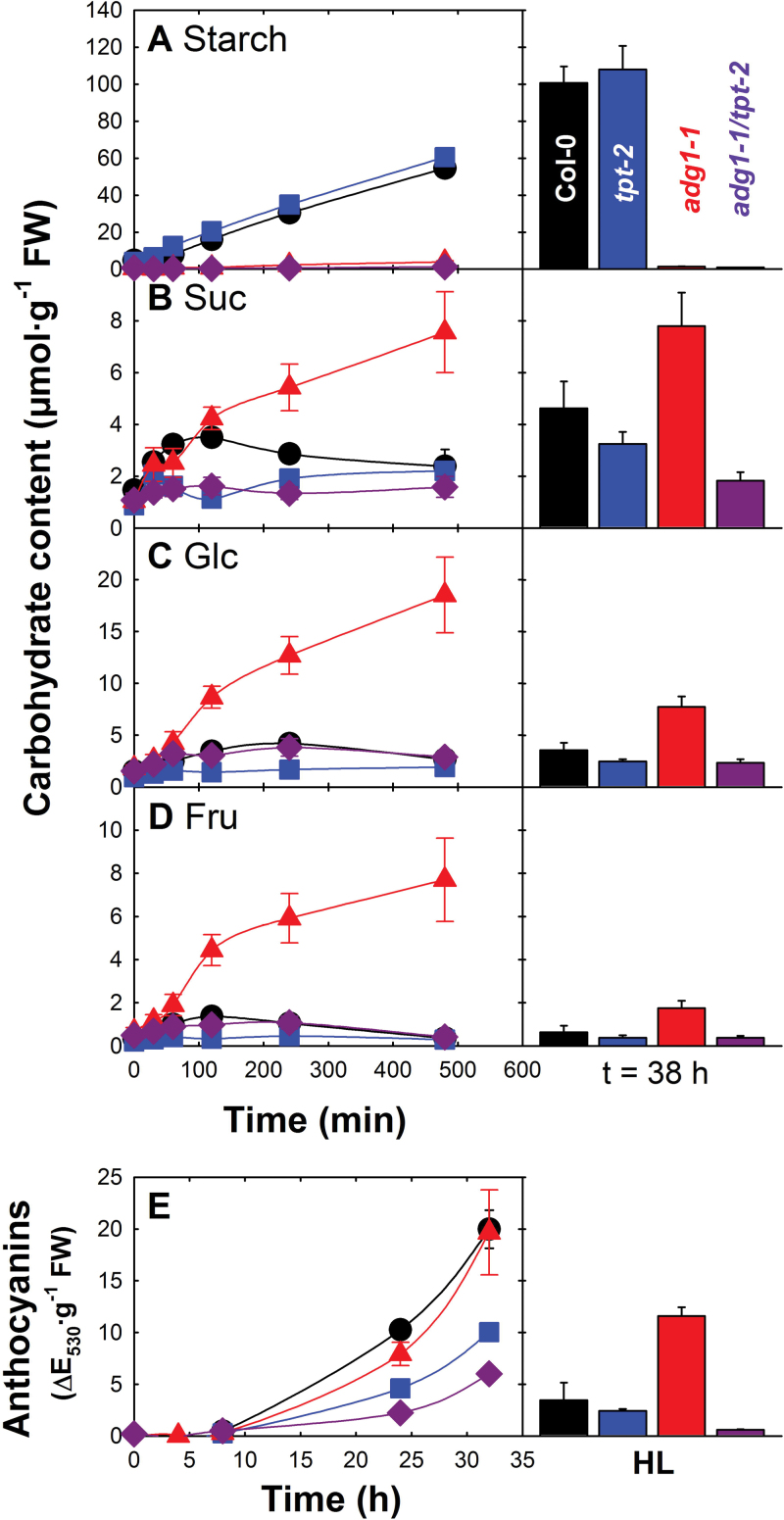
Short-term responses of leaf carbohydrate contents upon LL/HL transfer of wild-type and mutant plants. Col-0 (circles), *tpt-2* (squares), *adg1-1* (triangles), and *adg1-1/tpt-2* (diamonds) plants were grown for 3 weeks in LL under long-day conditions. The plants were transferred to HL at *t*=0 (4h in the light). The contents of starch (A), Suc (B), Glc (C), and Fru (D) have been monitored within the first 480min (8h; left panel) and after 38h (right panel). In (E), anthocyanin accumulation in wild-type and mutant plants is displayed during the first 32h after transfer from LL to HL and is compared with steady-state levels of anthocyanins in HL-grown plants. The data represent the mean ±SE of *n*=5 replicates. In some cases, the error bars are smaller than the symbols. (This figure is available in colour at *JXB* online.)

Dynamic changes in carbohydrate contents were assessed in wild-type and mutant plants within the first 8h, and 38h after LL/HL transfer (Fig. 2). Starch contents linearly increased with time only in wild-type and *tpt-2* mutant plants and approached levels of ~100 μmol g^–1^ fresh weight (FW) at the end of day 2 (i.e. after 38h; Fig. 2A). The soluble sugars sucrose (Suc), glucose (Glc), and fructose (Fru) dramatically increased in *adg1-1* within the first 8h and 38h after LL/HL transfer, but showed only a transient increase in wild-type, *tpt-2*, and *adg1-1/tpt-2* plants (Fig. 2B–D). Significant differences were observed for most time points only between *adg1-1* and the other lines (see Table 2 of Supplementary Document S1 available at *JXB* online). The transient accumulation of carbohydrates in all lines reflects an increased capacity to assimilate CO_2_ under HL conditions. However, in *adg1-1/tpt-2*, a prolonged time in HL led to a depletion of soluble sugars (compare Schmitz *et al.*, 2012).

**Table 2.** Enrichment of functional categories among the commonly regulated genes in wild-type and mutant plants as a response to LL/HL transfer at *t*_4h_ versus *t*_0_ and *t*_48h_ versus *t*_0_The threshold of log2 ratios was kept at ±1.5. Only functional categories with five or more altered genes are displayed.

Functional categories	No. of commonly regulated genes			
	*t*_4h_ versus *t*_0_		*t*_48h_ versus *t*_0_	
	Up-regulated	Down-regulated	Up-regulated	Down-regulated
Cell wall	–	16 (2.65)*	–	11 (3.05)*
Development	9 (1.12)	7 (0.85)	5 (2.58)	5 (1.02)
DNA synthesis/chromatin structure	9 (0.96)	–	–	
Hormone metabolism	5 (0.87)	13 (2.20)*	–	14 (3.96)*
Lipid metabolism	7 (1.54)	10 (2.15)*	–	7 (2.51)*
Major CHO metabolism	11 (8.87)*	–	–	–
Misc. – Cytochrome P450	5 (2.01)	–	–	–
Misc. – (UDP glucosyl- and glucoronyl transferases)	5 (2.48)	–	–	–
Protein – degradation	11 (0.59)*	21 (1.10)	–	10 (0.88)
Protein – post-translational modification	–	10 (1.18)	–	6 (1.18)
Protein – synthesis	8 (1.24)	–	–	–
Protein – targeting	6 (1.97)	–	–	–
RNA – processing	9 (2.89)*	–	–	–
RNA – regulation of transcription (RT)	30 (1.24)	44 (1.78)*	–	22 (1.49)
Secondary metabolism – flavonoids	5 (5.18)*	–	11 (47.32)*	–
Signalling	8 (0.55)*	21 (1.42)	–	15 (1.69)
Stress – abiotic	10 (2.16)*	9 (1.91)	–	5 (1.77)
Stress – biotic	6 (1.12)	–	–	–
Transport	14 (1.22)	18 (1.54)	7 (2.54)*	11 (1.57)

The numbers of significantly (*P*<0.01) altered genes were taken from Supplementary Table S7 available at *JXB* online.

The threshold of log2 ratios was kept at ±1.5.

The data in parentheses refer to over- or under-represented functional categories (i.e. numbers above or below 1).

Significantly (*P*<0.05) over- or under-represented categories according to the Fisher’s exact test are marked by an asterisk.

Over-represented categories are shown in bold letters, whereas under-represented categories are in italics.

LL/HL transfer also resulted in anthocyanin accumulation in wild-type and *adg1-1* plants (Fig. 2E), which was, however, less pronounced in *tpt-2* and *adg1-1/tpt-2*. Anthocyanin levels in HL-grown plants (most pronounced in *adg1-1*) appeared to be directly correlated with soluble carbohydrates (Fig. 2B–D, right panel). Suc is the most prominent inducer of anthocyanin production involving the MYB75/PAP1 (Teng *et al.*, 2005) and HY5 transcription factors (Shin *et al.*, 2013) as well as the COP1/SPA ubiquitin ligase (Maier *et al.*, 2013). For further statistical data analyses, see Table 2 of Supplementary Document S1 available at *JXB* online.

### *Lhcb1* expression and MgProtoIX levels were not correlated in response to LL/HL transfer

**Fig. 3.**
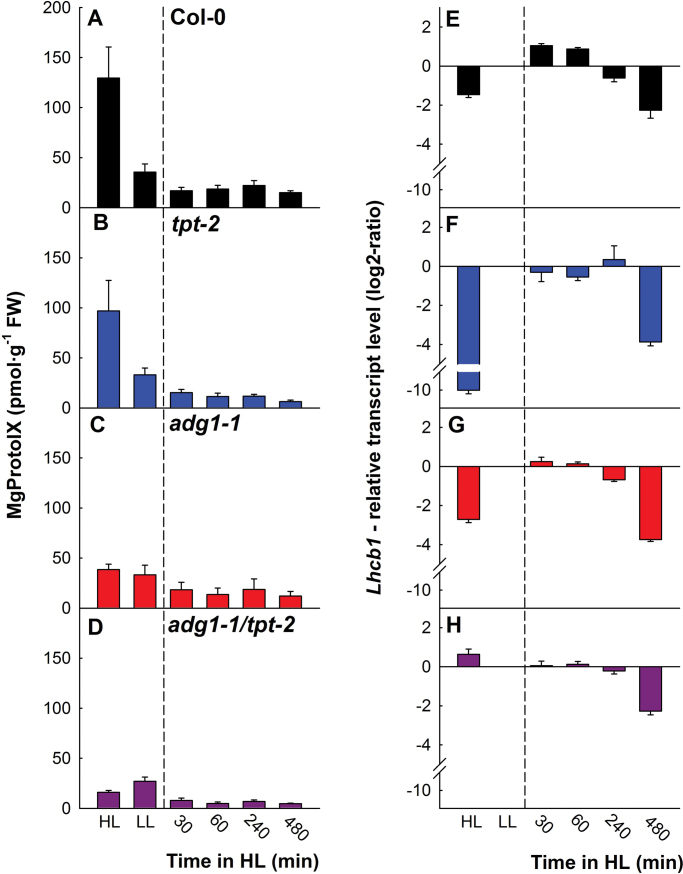
Contents of MgProtoIX (A–D) and transcript levels of *Lhcb1* (E–H) in Col-0 wild-type plants, the *tpt-2* and *adg1-1* single mutants, and the *adg1-1/tpt-2* double mutant grown either continuously in HL or LL or at different time intervals after LL/HL transfer. (This figure is available in colour at *JXB* online.)

The Chl biosynthesis intermediate, MgProtoIX, was proposed to act as a retrograde signal (Susek *et al.*, 1993; Strand *et al.*, 2003), although this idea has recently been challenged by more refined determinations of tetrapyrroles and the lack of correlation of their content with the abundance of *Lhcb1* transcripts (LHCII; Mochizuki *et al.*, 2008; Moulin *et al.*, 2008). Changes in MgProtoIX contents and *Lhcb1* expression levels in mutant and wild-type plants in response to short- and long-term HL exposure (Fig. 3) lacked any direct correlation (Supplementary Fig. S1A available at *JXB* online). In all plants, MgProtoIX initially dropped to lower levels upon HL exposure compared with LL-grown plants (Fig. 3A–D). Long-term HL exposure resulted in higher levels of MgProtoIX in wild-type and *tpt-2* plants (Fig. 3A, B), but not in *adg1-1/tpt-2* (Fig. 3D), and was less pronounced in *adg1-1* (Fig. 3C). Likewise, apart from small differences within the first 60min after LL/HL transfer, the short-term response of *Lhcb1* expression was similar between all plant lines (Fig. 3E–H). However, while *Lhcb1* expression was suppressed to a different degree in HL-grown wild-type and both single mutant plants (Fig. 3E–G), it was slightly induced in *adg1-1/tpt-2* (Fig. 3H). These data further indicate that acclimation to HL is perturbed in the double mutant. A statistical analysis of the data shown in Fig. 3 is given in Table 3 of Supplementary Document S1 available at *JXB* online.

**Table 3.** Pearson correlation coefficients calculated for comparisons of in-house with publicly available microarray dataIn (A) a list of commonly regulated genes (Supplementary Table S7 available at *JXB* online) and up-regulated genes in *tpt-2*, 48h after LL/HL transfer (Supplementary Table S8K* available at *JXB* online) have been used as basis for comparison. In (B) the gene lists for comparisons were obtained from Supplementary Tables S5A–C and S6A–C (available at *JXB* online).

Microarray data sets	A	*t*_4h_ all	*t*_48h_ all	*t*_48h_ tpt-2*	B	*t*_4h_ Col-0	*t*_48h_ Col-0	*t*_4h_ *adg1-1*	*t*_48h_ *adg1-1*	*t*_4h_ *tpt-2*	*t*_48h_ *tpt-2*	*t*_4h_ *adg1-1/tpt-2*	*t*_48h_ *adg1-1/tpt-2*
	Pearson correlation coefficient (0 to ±1)												
Seedlings+sucrose		**0.759**	**0.901**	–0.479		ND	ND	ND	ND	ND	ND	ND	ND
*pho3* mutant		**0.866**	**0.956**	0.191		**0.976**	None	0.357	0.601	0.627	0.322	**–0.933**	None
ABA feeding		–0.093	0.242	0.238		–0.026	0.692	0.299	–0.049	0.556	0.247	–0.607	–0.107
IAA feeding		–0.278	0.061	0.176		0.282	**0.735**	–0.063	–0.514	None	0.129	–0.407	–0.217
Zeatin feeding		0.110	–0.089	0.251		–0.369	**–0.991** ^*a*^	–0.722	–0.230	–0.589	0.140	0.386	–0.223
GA_3_ feeding		0.249	–0.527	**0.808**		None	None	0.136	0.641	None	0.190	None	None
ACC feeding		–0.232	0.178	0.533		0.375	0.520^*a*^	0.605	0.061	None	0.026	0.650	None
MJ feeding		0.600	0.609	0.279		**0.874**	**0.795**	0.113	0.345	–0.589	0.170	–0.234	–0.256
*flu* mutant+light		0.374	0.063	–0.077		0.317	–0.497	0.140	0.041	**–0.863**	0.353	–0.045	–0.090
Wild type+MV (24h)		0.476	**0.725**	0.516		ND	ND	ND	ND	ND	ND	ND	ND

The colums in (A) refer to threshold log2-fold change of ±1.5, whereas the columns in (B) are based on a threshold log2 ratio of ±1.0. Pearson correlation coefficients above/below 0.7/–0.7 are displayed in bold.

ND, not determined; none, less than three commonly regulated genes.

^*a*^ Less than four genes.

Although MgProtoIX did not correlate with *Lhcb1* expression, there was a pronounced inverse correlation of *Lhcb1* transcript levels with soluble carbohydrate contents in wild-type and mutant plants (Supplementary Fig. S1E available at *JXB* online). Hence, carbohydrates might play a profound role in the transcriptional acclimation response of *A. thaliana*. In contrast, MgProtoIX levels were directly correlated with the light reaction of photosynthesis (i.e. ETR; Supplementary Fig. S1F available at *JXB* online) rather than with products of the dark reaction (Supplementary Fig. S1B, C available at *JXB* online).

### Redox components were similarly affected in mutant and wild-type plants after LL/HL transfer

Light stress leads to the production of ROS, which can cause severe damage to membranes and the photosynthetic apparatus (Apel and Hirt, 2004; Vass, 2012). Besides ROS with a short lifetime such as singlet oxygen (Krieger-Liszkay, 2005), those with a longer lifetime suh as, for instance, H_2_O_2_, would have the potential to leave the chloroplast and hence to act as a retrograde signal (Noctor *et al.*, 2000; Karpinski *et al.*, 2013). As H_2_O_2_ can also be produced in the peroxisomes during photorespiration and in the apoplast, the mesophyll has to distinguish between different sources of H_2_O_2_ signals (Shapiguzov *et al.*, 2013).

**Fig. 4.**
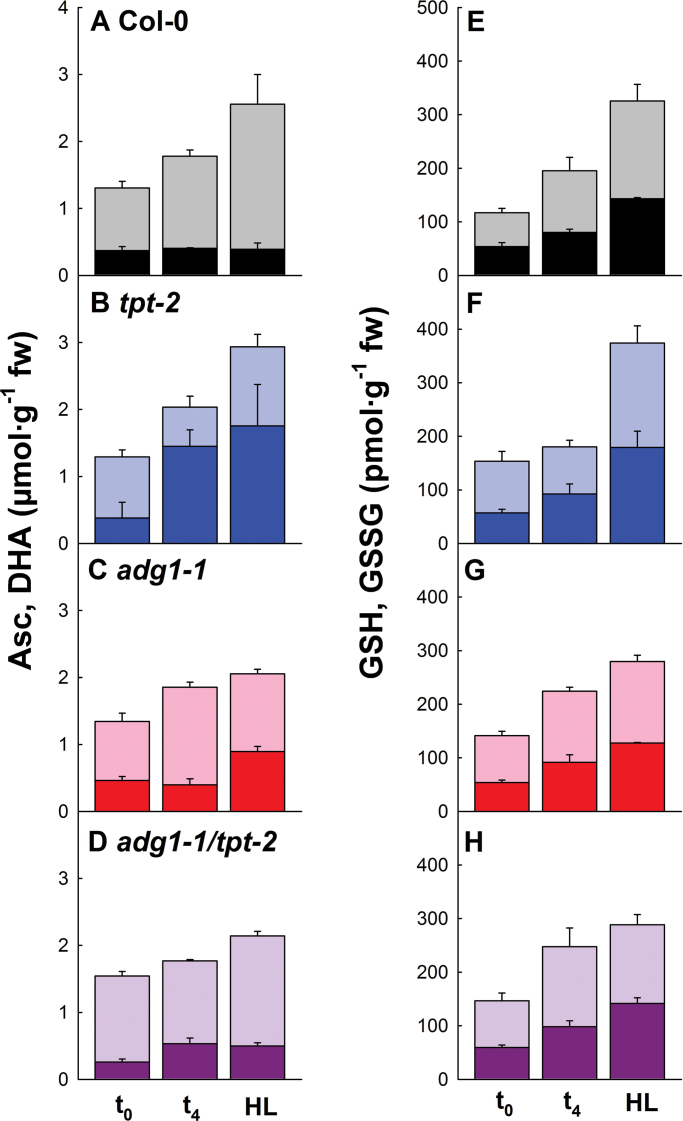
Contents of redox components in Col-0 wild-type (A, E), *tpt-2* (B, F) and *adg1-1* (C, G) single mutants, and *adg1-1/tpt-2* (D, H) double mutant plants grown in LL (*t*_0_) or HL, or 4h after LL/HL transfer (*t*_4h_). The reduced compounds of the ascorbate (Asc)/dehydroascorbate (DHA) system (D) and the glutathione (GSH)/glutathione disulphide (GSSG) system (E–H) are represented by the lighter portions of the bars, whereas the oxidized compounds are marked by the darker portions. The data represent the mean ±SE of 3–5 replicates. (This figure is available in colour at *JXB* online.)

The major H_2_O_2_ source in photosynthesis is the Mehler–peroxidase reaction or ‘pseudo cyclic’ electron transport (Badger *et al.*, 2000). H_2_O_2_ can be detoxified by either the Asc–GSH cycle (Halliwell–Asada pathway; Noctor and Foyer, 1998) or the Asc-independent thiol peroxidase pathway (Dietz *et al.*, 2006). Asc and DHA as well as GSH and GSSG levels after LL/HL transfer lack large differences in *adg1-1/tpt-2* (Fig. 4D, H) compared with wild-type plants (Fig. 4A, E). All plant lines show an increase in the total contents of both redox components depending on the time in HL. Significant changes in Asc and DHA levels were observed in the *tpt-2* mutant, which resulted in a drop of the Asc/DHA ratios below one (Fig. 4B). This was, however, not reflected in large changes in GSH/GSSG ratios (Fig. 4F).

Although the exposure of LL-grown *adg1-1/tpt-2* plants to HL resulted in severe photoinhibition, the redox components involved in the detoxification of ROS were hardly altered, most probably because photoinhibition also restricts the production of ROS at the thylakoid membrane. Hence, differential redox signalling as a cause of the phenotype of *adg1-1/tpt-2* appears to be unlikely. For statistical analyses, see Table 4 of Supplementary Document S1 available at *JXB* online.

**Table 4.** Contents of selected metabolites determined either enzymatically (starch, d-Suc, d-Glc, and d-Fru) or by GC-MS in leaf extracts of (A) Col-0 and *tpt-2* or (B) *adg1-1* and *adg1-1/tpt-*2 grown either continuously in LL or HL or after LL/HL transfer at *t*_4h_ or *t*_48h_

A								
Metabolites	Col-0 *t*_0_ (LL)	Col-0 *t*_4h_	Col-0 *t*_48h_	Col-0 (HL)	*tpt-2 t*_0_ (LL)	*tpt-2 t*_4h_	*tpt-2 t*_48h_	*tpt-2* (HL)
	Starch and sugar content (μmol g^–1^ FW)							
Starch	2.86±0.29	33.04±1.30	70.81±3.66	122.50±7.19	5.33±0.23	36.93±5.60	110.17±3.74	148.49±5.97
d-Suc	0.77±0.06	1.97±0.04	2.66±0.25	3.90±0.45	0.60±0.03	1.06±0.02	3.07±0.20	1.70±0.23
d-Glc	1.02±0.12	2.05±0.12	2.69±0.25	8.32±1.06	0.95±0.05	1.14±0.06	3.50±0.22	5.95±0.86
d-Fru	0.10±0.01	0.56±0.02	0.57±0.06	1.69±0.21	0.01±0.01	0.12±0.02	0.53±0.07	1.20±0.13
	Relative metabolite content, GC-MS (arbitrary units g^–1^ FW)							
**Sugars**								
d-Suc	3.26±0.06	6.08±0.10	7.33±0.12	6.78±0.19	3.39±0.06	4.18±0.16	7.15±0.13	8.13±0.25
d-Glc	0.53±0.04	21.35±0.68	12.21±1.14	13.71±1.25	0.67±0.04	3.19±0.22	22.97±0.69	16.01±0.75
d-Fru	0.74±0.04	16.80±0.45	9.68±1.85	12.78±0.90	0.91±0.03	3.38±0.24	17.42±0.46	8.61±0.56
d-Maltose	0.12±0.01	0.25±0.02	1.87±0.26	0.52±0.03	0.89±0.05	0.51±0.06	10.98±0.81	6.71±0.95
α,α′-d-Trehalose	0.25±0.03	0.48±0.03	4.10±0.43	2.84±0.17	0.25±0.02	0.31±0.03	1.22±0.13	0.84±0.08
**Amino acids**								
dl-Glutamic acid	32.46±1.86	124.73±2.57	165.01±2.40	39.41±3.02	53.29±2.59	98.74±6.50	169.43±3.91	87.26±9.74
Glycine	3.52±0.23	144.51±2.41	115.60±1.32	14.48±0.33	4.41±0.26	107.48±1.70	109.62±2.31	80.02±3.37
dl-Serine	10.12±0.36	28.05±0.75	54.53±1.41	38.38±0.84	15.97±0.71	65.01±0.91	66.14±1.24	63.03±1.45
l-Proline	7.16±0.83	35.89±1.73	60.06±3.78	14.33±2.1	6.84±0.43	70.17±4.47	42.50±4.54	70.52±5.59
**Organic acids**								
Pyruvic acid	0.61±0.03	0.67±0.06	0.97±0.07	0.84±0.05	0.79±0.06	1.64±0.18	1.07±0.04	1.61±0.19
2-Methyl-dl-malic acid	0.09±0.01	0.34±0.03	1.02±0.03	0.51±0.03	0.17±0.01	0.42±0.02	1.21±0.04	0.56±0.05
Fumaric acid	106.36±3.90	125.30±1.85	111.55±2.94	133.31±2.25	117.78±3.67	101.63±2.49	91.51±2.35	112.65±3.50
**Others**								
Glycerol	1.50±0.07	2.97±0.30	2.91±0.14	3.99±0.47	1.42±0.05	2. 79±0.24	2.41±0.13	3.55±0.18
myo-Inositol	12.82±0.58	19.92±0.40	23.73±1.37	26.92±0.37	11.44±0.72	18.02±0.44	23.18±0.54	15.78±0.56
Erythritol	0.27±0.01	0.73±0.09	1.06±0.07	0.82±0.05	0.35±0.03	0.78±0.06	1.28±0.07	1.29±0.16
Putrescine	1.09±0.07	7.53±0.64	10.94±0.94	3.11±0.49	1.14±0.05	3.90±0.35	16.55±1.00	14.76±1.63
**B**								
**Metabolites**	***adg1-1 t***_**0**_ **(LL)**	***adg1-1 t***_**4h**_	***adg1-1 t***_**48h**_	***adg1-1*** **(HL)**	***adg1-1/ tpt-2 t***_**0**_ **(LL)**	***adg1-1/ tpt-2 t***_**4h**_	***adg1-1/ tpt-2 t***_**48h**_	***adg1-1/ tpt-2*** **(HL)**
	Starch and sugar content (μmol g^–1^ FW)							
Starch	0.27±0.01	0.52±0.03	0.67±0.06	0.11±0.06	0.42±0.03	0.85±0.03	0.68±0.04	1.82±0.79
d-Suc	1.74±0.14	3.90±0.62	8.67±0.87	3.43±0.74	1.34±0.16	1.52±0.10	1.99±0.25	1.02±0.09
d-Glc	2.40±0.14	11.11±0.08	7.89±0.56	9.65±0.58	2.05±0.08	3.60±0.04	4.00±0.20	3.61±0.15
d-Fru	0.89±0.10	6.04±0.16	2.68±0.29	3.11±0.16	0.66±0.01	0.98±0.06	0.78±0.10	0.85±0.06
	Relative metabolite content, GC MS (arbitrary units g^–1^ FW)							
**Sugars**								
d-Suc	5.66±0.18	6.69±0.06	7.08±0.05	7.90±0.12	3.79±0.15	5.20±0.07	5.93±0.08	4.77±0.19
d-Glc	6.77±0.53	25.97±0.17	24.41±0.44	26.82±0.37	2.92±0.44	13.41±1.30	10.77±0.74	5.73±0.77
d-Fru	10.91±0.63	20.17±0.21	19.71±0.37	20.95±0.34	3.92±0.48	5.84±0.47	8.44±0.54	3.76±0.29
d-Maltose	ND	0.55±0.03	0.52±0.04	0.14±0.01	ND	0.38±0.04	0.21±0.02	0.14±0.03
α,α′-d-Trehalose	0.16±0.01	0.44±0.03	3.33±0.23	1.48±0.07	0.15±0.02	0.18±0.02	0.25±0.03	0.76±0.10
**Amino acids**								
dl-Glutamic acid	29.83±1.81	65.86±5.49	176.49±1.89	37.77±1.71	35.30±1.80	73.15±5.63	70.01±4.78	37.78±4.50
Glycine	4.78±0.24	84.63±3.23	116.06±1.69	22.94±1.59	2.52±0.15	20.29±1.63	7.62±0.38	4.30±0.26
dl-Serine	6.10±0.22	23.16±1.38	49.68±1.48	25.88±1.76	7.70±0.52	19.02±1.19	16.56±0.49	14.93±0.68
l-Proline	4.01±0.29	30.16±2.26	68.00±7.02	18.81±2.64	6.94±1.31	26.60±1.85	22.41±4.80	9.86±1.32
**Organic acids**								
Pyruvic acid	0.53±0.04	0.53±0.04	0.87±0.05	1.09±0.06	0.62±0.03	1.10±0.07	1.09±0.12	0.62±0.02
2-Methyl-dl-malic acid	0.12±0.01	0.36±0.03	1.13±0.06	0.62±0.02	0.16±0.02	0.38±0.01	0.62±0.02	0.16±0.02
Fumaric acid	136.96±4.41	115.53±1.70	98.82±3.49	116.77±2.31	96.78±1.61	91.04±1.66	85.79±1.36	82.43±10.17
**Others**								
Glycerol	1.24±0.02	2.18±0.25	2.55±0.05	4.42±1.15	1.22±0.13	2.27±0.16	2.33±0.19	10.11±3.54
myo-Inositol	6.35±0.35	13.79±0.78	18.83±0.74	16.17±0.54	7.07±0.16	13.69±0.51	16.35±0.44	6.16±0.35
Erythritol	0.53±0.04	1.07±0.13	4.69±0.38	1.44±0.10	0.89±0.02	1.57±0.09	1.80±0.04	1.96±0.21
Putrescine	2.93±0.10	9.60±1.26	36.15±2.69	11.37±1.12	1.92±0.12	6.92±0.98	5.18±0.39	5.95±0.88

The samples were harvested in the middle of the light period (i.e. after 8h in the light).

The data represent the mean of five independent samples ±SE; ND, not detected.

### Assessment of transcriptomic changes in response to LL/HL transfer of mutants impaired in carbohydrate metabolism

The role of impaired carbon partitioning in HL acclimation has been assessed in wild-type and mutant plants by microarray-based transcriptomics in time series upon LL/HL transfer. Suitable time points for sampling were obtained from experiments shown in Figs 1 and 2, and were LL (*t*_0_), as well as 4h (*t*_4h_; as short-term response) and 48h (*t*_48h_; as long-term response) after transfer to HL. At the latter time point, the ETR in wild-type and single mutant plants started to increase in response to HL exposure (Fig. 1B).

**Fig. 5.**
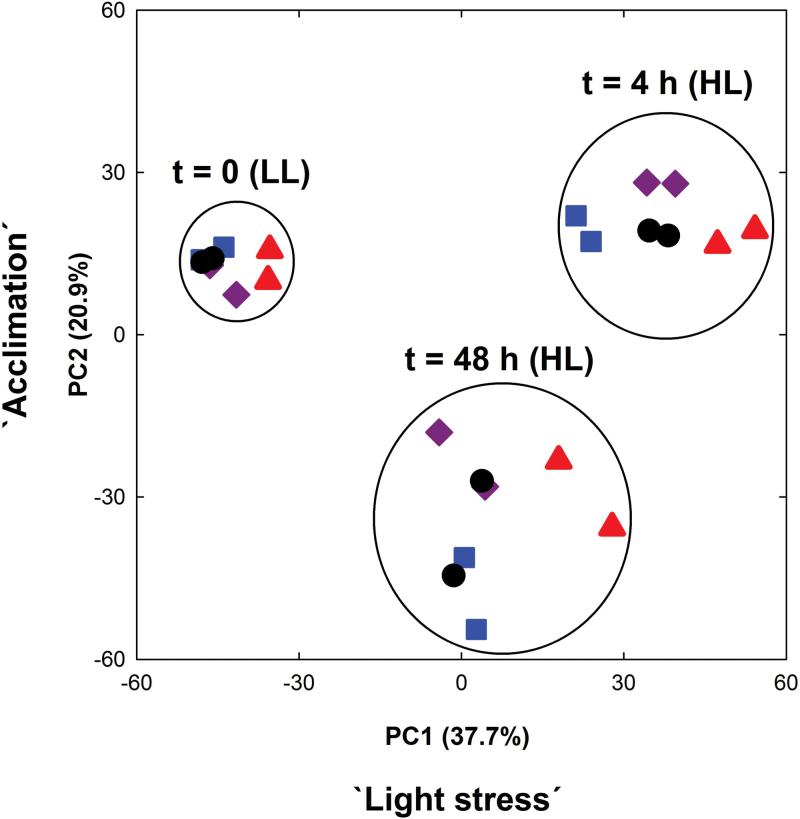
Multivariate analysis (principle components) of the microarray data sets of LL/HL transfer experiments with Col-0 wild-type (circles), the *tpt-2* (squares) and *adg1-1* (triangles) single mutants, and the *adg1-1/tpt-2* double mutant (diamonds). (This figure is available in colour at *JXB* online.)

**Fig. 6.**
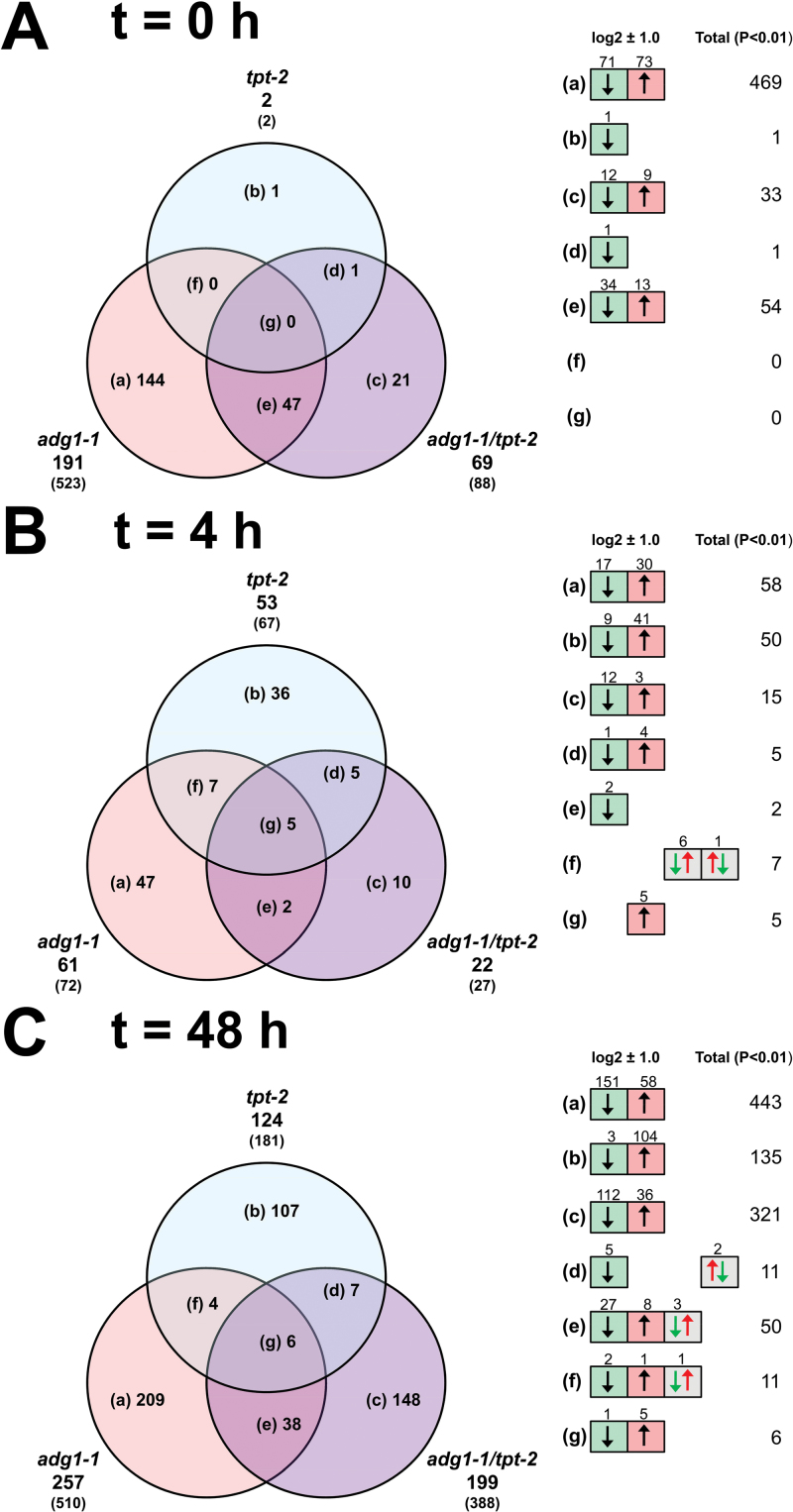
Venn diagrams showing ‘static’ comparisons of differentially regulated genes between mutant and wild-type plants at *t*=0h (A), *t*=4h (B), and *t*=48h (C). Genes that were significantly (*P*<0.01) altered at a threshold of log2 ratios of ±1.0 are displayed. The numbers in parentheses and in the right panel represent total numbers of significantly altered genes without any threshold. (This figure is available in colour at *JXB* online.)

**Fig. 7.**
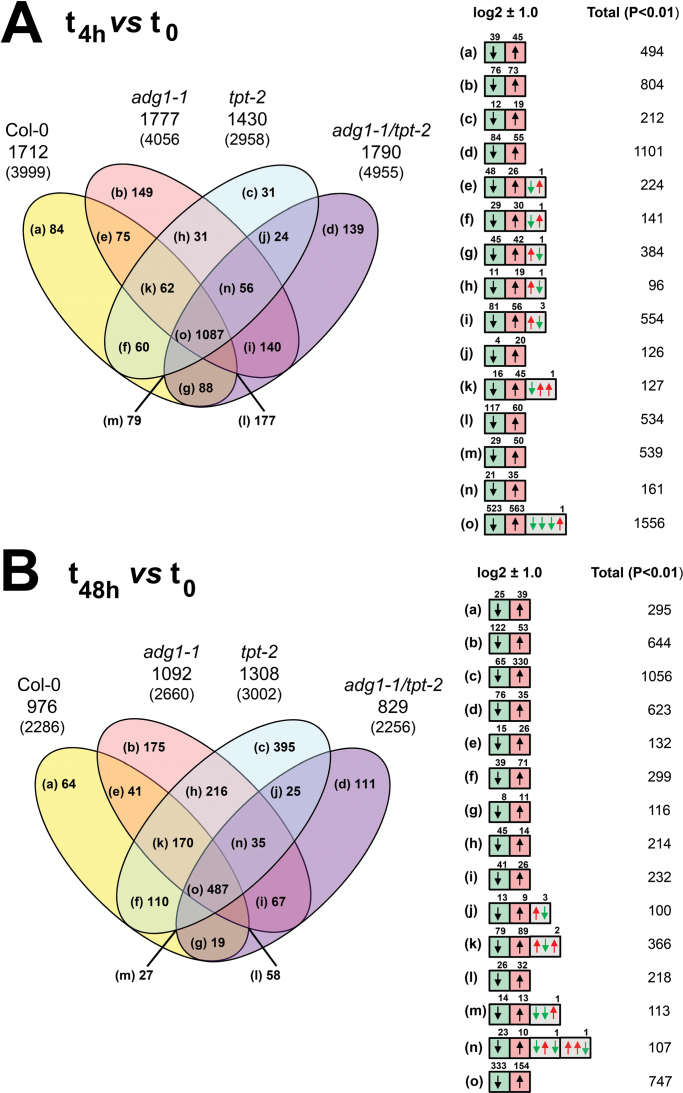
Venn diagrams showing ‘dynamic′ comparisons of differentially regulated genes in wild-type and mutant plants at *t*_4h_ versus *t*_0_ (A) and *t*_48h_ versus *t*_0_ (B). Genes that were significantly (*P*<0.01) altered at a threshold of log2 ratios of ±1.0 are displayed. The numbers in parentheses and in the right panel represent total numbers of significantly altered genes without any threshold. The number of genes differently regulated at *t*_4h_ (A) and *t*_48h_ (B) in HL compared with *t*_0_ in LL are displayed. (This figure is available in colour at *JXB* online.)

A multivariate analysis of the data sets (PCA; significance level, *P*>0.01; no threshold) revealed three individual clusters that were governed by the light regime and/or the time in HL rather than by metabolic defects of the respective mutants (Fig. 5). The microarray data were further analysed by ‘static’ and ‘dynamic’ comparisons of gene expression. For the ‘static’ approach (for details, see Supplementary Document 2 available at *JXB* online), expression levels in the mutants were referred to wild-type plants at each time point. Numbers of significantly altered genes are shown in the Venn diagrams (Fig. 6) and in more detail in Supplementary Tables S2–S4 available at *JXB* online. For a dynamic assessment of HL-responsive genes in wild-type and mutant plants, their expression level at *t*_4h_ and *t*_48h_ after LL/HL transfer were referred to *t*_0_ (Fig. 7; Supplementary Tables S5, S6 available at *JXB* online).

### Static assessment of global gene expression after LL/HL transfer

Under LL conditions, 523 genes were significantly altered in *adg1-1* compared with 88 in *adg1-1/tpt-2* and only two in *tpt-2* (Fig. 6A). After 4h in HL, the number of differentially regulated genes dropped to lower values in *adg1-1* and *adg1-1/tpt-2* (Fig. 6B), but increased again after 48h of HL exposure (Fig. 6C). In *tpt-2*, the number of altered genes increased from 67 to 181 after 4h and 48h, respectively, in HL. However, the highest number was again found in *adg1-1* (510) rather than in *adg1-1/tpt-2* (388). The number of commonly regulated genes in the central overlapping regions of the Venn diagrams was small, suggesting that the alterations in gene expression compared with wild-type plants observed in the ‘static approach’ were related to the individual lesions in the mutants rather than to changing conditions. Static changes in expression of HL-responsive genes have been analysed and described in more detail in Supplementary Document S2 and in Supplementary Tables S2–S4 available at *JXB* online. Functional categories containing more than five genes are shown in Table 1. In *adg1-1/tpt-2*, significantly over-represented genes of the categories ‘amino acid metabolism’, ‘cell wall’, and ‘CHO metabolism’ were down-regulated at *t*_48h_ after LL/HL transfer, whereas over-represented plastome-encoded genes belonging to the categories ‘protein – synthesis′ and ‘PS – light reaction’ were up-regulated. Remarkably, in *adg1-1/tpt-2*, MYC4 (At4g17880), a basic helix–loop–helix (bHLH)-type transcription factor involved in jasmonate responses (Fernández-Calvo *et al.*, 2011) and glucosinolate biosynthesis (Schweizer *et al.*, 2013), was severely down-regulated in both LL and HL. According to the eFP browser (Winter *et al*., 2007), *MYC4* is also inducible by sugars.

### Dynamic changes in gene expression during LL/HL transfer experiments

Compared with the static assessment of global gene expression, which aimed at identifying differentially regulated genes between mutant and wild-type plants at each time point, the dynamic analysis displayed genes particularly responding to the LL/HL transfer and is therefore described in more detail.

Interestingly, 4h after HL exposure, the overlapping areas of all plants in this study comprised the highest number (1087) of significantly altered genes (Fig. 7A), which also showed a relatively high amplitude of differential expression (i.e. log2 ratios of ±3; Supplementary Table S5 available at *JXB* online). This observation indicates that the short-term response of HL-dependent regulation of a large set of genes was operational in wild-type and mutant plants. After 48h in HL, the number of genes in the central overlapping region of the Venn diagrams dropped by ~50% compared with *t*_4h_ (Fig. 7B; Supplementary Table S7B available at *JXB* online), but increased specifically in *tpt-2* from 31 to 395 genes at *t*_4h_ and *t*_48h_, respectively. Compared with *tpt-2*, the number of altered genes was appreciably smaller in *adg1-1* (175) and *adg1-1/tpt-2* (111).

### Strategies for functional analyses of HL-responsive genes

Different strategies were applied to obtain additional information on HL-responsive genes in wild-type and mutant plants. The following observations and assumptions have been addressed. (i) It can be assumed that commonly regulated HL-responsive genes in all lines of this study should be independent from metabolic constraints by individual mutations. (ii) In contrast, specifically regulated or non-regulated genes after LL/HL transfer should be linked to the metabolic constraints in the individual mutant plants. Finally, (iii) comparisons of in-house with publicly available microarray data on hormone and stress treatments might help to identify components associated with signalling pathways involved in HL acclimation.

### Wild-type and mutant plants comprise a large set of commonly regulated genes in response to LL/HL transfer

The central regions of the Venn diagrams shown in Fig. 7 comprise genes responding similarly to HL exposure in wild-type and mutant plants and can hence be considered as independent or less dependent than lesions in primary carbohydrate metabolism. These genes are listed in Supplementary Table S7 available at *JXB* online. The number of genes was narrowed down by adjusting the threshold log2 ratio to ±1.5. Under HL conditions, 562 and 237 genes were differentially regulated 4h and 48h after LL/HL transfer, respectively (Supplementary Table S7A, B available at *JXB* online). The distribution of functional categories comprising five or more genes per category is shown in Table 2.

Strikingly, the diversity of functional categories was larger at *t*_4h_ versus *t*_0_ compared with *t*_48h_ versus *t*_0_. In particular, the number of categories containing up-regulated genes was higher at *t*_4h_ (i.e. 17 categories) compared with *t*_48h_ (i.e. three categories) after LL/HL transfer. In both cases the category ‘unknown’ has been excluded. At *t*_4h_ versus *t*_0_, amongst the over-represented categories ‘major CHO metabolism’, ‘secondary metabolism – flavonoids’, ‘RNA processing’, and ‘abiotic stress’ were up-regulated, whereas ‘cell wall’, ‘hormone’, ‘lipid metabolism’, and ‘regulation of transcription (RT)’ were down-regulated (Table 2). With the help of the above approach, it was possible to dissect the data into a large variety of short-term, HL-responsive genes and a limited number of categories that respond in the long term to HL exposure (Table 2).

Genes associated with ‘RT’, ‘signalling’, ‘abiotic stress’, or ‘biotic stress’, as well as ‘hormone metabolism’ can be considered as candidates that might be involved in retrograde signalling. There were 30 genes belonging to the ‘RT’ category up-regulated and 45 down-regulated at *t*_4h_ versus *t*_0_ compared with four up- and 22 down-regulated genes at *t*_48h_ versus *t*_0_. Strikingly, none of the up-regulated and only 13 of the down-regulated RT genes was found at both *t*_4h_ and *t*_48h_ upon HL exposure, suggesting that this dynamic response occurred only transiently (Supplementary Table S7 available at *JXB* online).

Likewise, the expression of genes belonging to the categories ‘signalling’ as well as ‘abiotic stress’ or ‘biotic stress’ was altered to a larger extent at *t*_4h_ compared with *t*_48h_, and, again, only a small number of genes belonging to these categories was found at both *t*_4h_ and *t*_48h_. The category ‘hormone metabolism’ comprises genes coding for response regulators as well as metabolic enzymes. At *t*_4h_ versus *t*_0_, this category contained a broad spectrum of phytohormones, such as ABA, gibberellic acid, brassinosteroids, cytokinins, and jasmonate, whereas at *t*_48h_ versus *t*_0_, 13 of the 14 down-regulated genes of the ‘hormone’ category were confined to auxins and only one to ethylene. For further information, see Supplementary Table S7 available at *JXB* online.

### Primary carbon metabolism and transport were commonly affected in the short term after HL exposure in all lines

A number of metabolic genes associated with ‘lipid metabolism’, ‘major CHO metabolism’, and ‘transport’ were differentially regulated as a response to LL/HL transfer, specifically in the short term, namely at *t*_4h_ (Table 2). The detailed analysis of these genes, which is contained in Supplementary Document S3 available at *JXB* online, can be summarized as follows: most of the 11 genes associated with ‘major CHO metabolism’ are involved in starch metabolism (see also Walters *et al.*, 2004). The transcriptional regulation of these genes is obviously independent from the presence or absence of starch, as it also works in the *adg1-1* background. An analysis with ATTEDII (Obayashi *et al.*, 2009) revealed that these genes are imbedded in a single regulatory network (Supplementary Fig. S2A, Supplementary Table S7A available at *JXB* online), which also comprises a chloroplast-localized AMP-activated protein kinase (At5g39790; Supplementary Table S7A available at *JXB* online). There appears to be a cross-talk between ‘CHO metabolism’ and ‘lipid metabolism’ as three members of this network are also contained in a network of up-regulated genes associated with ‘lipid metabolism’ (Supplementary Fig. S2B available at *JXB* online). Likewise, down-regulated genes involved in ‘lipid metabolism’ at *t*_4h_ versus *t*_0_ (but not at *t*_48h_ versus *t*_0_) form a large network (Supplementary Fig. S2C available at *JXB* online).

Inter- and intracellular transport processes are of particular importance in the adjustment of metabolism to the requirement of the plants. Among 32 differentially regulated genes associated with ‘transport processes’, the expression of two genes belonging to the phosphate translocator family was altered after 4h in HL. The glucose 6-phosphate/phosphate translocator2 (GPT2; At1g61800) and the phosphoenolpyruvate/phosphate translocator2 (PPT2; At3g01550) were up- and down-regulated, respectively. Interestingly, *GPT2* strongly responds to elevated soluble sugar levels (Kunz *et al.*, 2010; Schmitz *et al.*, 2012), for example in starch-free mutants or after feeding of exogenous carbohydrates to the plants (Heinrichs *et al.*, 2012). Although *GPT2* was highly up-regulated at *t*_4h_ versus *t*_0_ in all plants (Supplementary Table S7A available at *JXB* online), its expression level at *t*_48h_ versus *t*_0_ corresponded well to the sugar levels in the individual plant lines; that is, *GPT2* expression was further increased in *adg1-1*, but was not significantly changed in the double mutant (compare Fig. 1). For more information, please refer to Supplementary Document S3 available at *JXB* online.

### Does the mutant-specific regulation or non-regulation of genes provide information on missing signals?

Here the question was asked of whether the analysis of genes specifically regulated or not regulated in the individual mutant plants in response to LL/HL transfer can provide information on the putative retrograde signals involved.

**Fig. 8.**
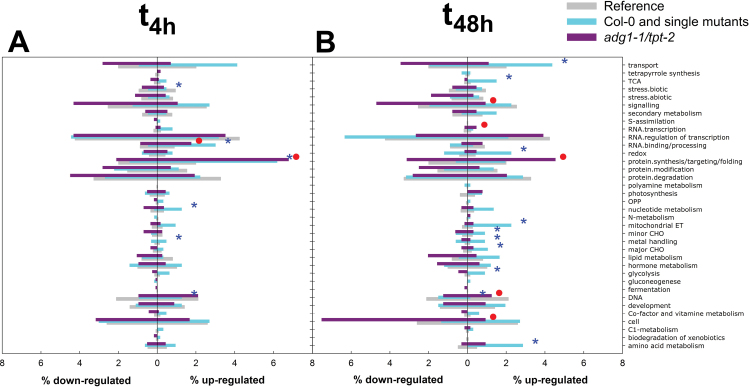
MapMan binning-based distribution of up- and down-regulated genes in individual functional categories 4h (A) and 48h (B) after LL/HL transfer. The reference value (light grey bars) refers to the number of genes belonging to a certain category expressed as a percentage of all genes on the microarray, whereas intermediate grey and dark grey bars refer to the number of up- or down-regulated genes belonging to a certain category as a percentage of all differentially regulated genes. Intermediate grey bars refer to all genes not regulated in *adg1-1/tpt-2* (i.e. genes regulated in Col-0 and the single mutants) and dark grey bars refer to genes specifically regulated in *adg1-1/tpt-2*. Significant differences (*P*<0.05) in the relative distribution amongst the individual categories were estimated by Fisher’s exact test and marked by either asterisks or circles for intermediate grey or dark grey bars, respectively. (This figure is available in colour at *JXB* online.)

In a first approach, the intention was to identify significantly (Fisher’s exact test; *P*<0.05) over- or under-represented functional groups or clusters according to MapMan bins (Thimm *et al.*, 2004). Significantly altered (*P*<0.01) HL-responsive or non-responsive genes in the *adg1-1/tpt-2* double mutant (i.e. those genes responding to HL only in wild-type and single mutant plants) were categorized without threshold (compare Fig. 7). The major outcome of this approach is shown in Fig. 8 and can be summarized as follows: the MapMan binning of the expression data resulted in 36 major functional categories. In the short term (i.e. 4h after LL/HL transfer; Fig. 8A), genes belonging to the categories ‘RNA binding/processing’ and ‘protein synthesis/targeting/folding’ were significantly over-represented in both groups, regulated and non-regulated genes, in *adg1-1/tpt-2.* All other significant alterations can be attributed to genes that were not differentially expressed in *adg1-1/tpt-2*, and these were the under-represented categories ‘biotic stress’ and ‘DNA’ as well as the over-represented categories ‘nucleotide metabolism’ and ‘metal handling’. In the long term (i.e. 48h after LL/HL transfer; Fig. 8B), the pattern of over- or under-represented functional categories was different compared with *t*_4h_. Only three out of 36 functional categories were specifically altered in the double mutant according to their relative abundance. Interestingly, in the categories ‘signalling’ and ‘cell’, down-regulated genes were over-represented (Fig. 8B), whereas the category ‘RNA – transcription’ contained over-represented up-regulated genes. There were also some interesting changes in the relative abundance of genes that were specifically not regulated in *adg1-1/tpt-2*. Nine out of the 36 functional categories contained over-represented up-regulated genes in Col-0 and the single mutants related to ‘transport’, ‘tricarboxylic acid cycle’, ‘redox’, ‘mitochondrial electron transport’, ‘minor CHO metabolism’, ‘major CHO metabolism’, ‘metal handling’, ‘glycolysis’, and ‘amino acid metabolism’, suggesting that these processes are less responsive in the double mutant.

In a second approach, genes specifically responding to the constraints in the single and double mutants were separated and are listed in Supplementary Table S8 available at *JXB* online. For this purpose, the log2-fold change for genes assigned as non-regulated was limited to ±0.5. In particular, *adg1-1/tpt-2* exhibited a strong phenotype upon HL exposure and hence further attention was focused on this line (compare MapMan binning, Fig. 8). At *t*_4h_ versus *t*_0_ *adg1-1/tpt-2* contained five up- and nine down-regulated genes (Supplementary Table S8A available at *JXB* online). Three of the five up-regulated genes were associated with histone proteins and hence ‘chromatin structure’ and ‘cell organization’. Besides a gene of unknown function (At2g30600), a methylthioalkylmalate synthase gene (At5g23010) involved in methionine-derived glucosinolate biosynthesis (Textor *et al.*, 2004) was up-regulated. Interestingly, of the nine down-regulated genes, four genes were associated with ‘biotic stress’ (three of these genes encode heat shock proteins; At1g53540, At3g46230, and At5g12020, which have also been recognized in the static approach; compare Supplementary Document S2 available at *JXB* online) and one gene each was linked to ‘calcium signalling’ (At2g41090), ‘RT’ (At5g61010), ‘mitochondrial electron transport’ (At4g05020), ‘minor CHO metabolism’ (At2g37760), and ‘lipid metabolism’ (At2g44300). In particular, heat shock proteins represent the interaction point of various stress response pathways (Swindell *et al.*, 2007) and deserve further attention also with respect to retrograde signalling. In the category ‘RT’ an Exo70 type subunit of an exocytosis complex was down-regulated. Such organelle-like structures are involved in excretion processes by fusion with the plasma membrane (Li *et al.*, 2010; Wang *et al.*, 2010).

At *t*_48h_ versus *t*_0_ there were five and six genes up- or down-regulated specifically in *adg1-1/tpt-2* (Supplementary Table S8C available at *JXB* online). Amongst the up-regulated genes, plastome-encoded ribosomal proteins (two genes) and one gene encoding RNA polymerase were found. The further two up-regulated genes were associated with either ‘amino acid degradation’ (At3g16150) or ‘RT’, namely the heat shock transcription factor A9 (At2g26150), which under normal conditions is only weakly expressed in leaves (eFP browser, Winter *et al.*, 2007), again bringing members of heat shock proteins or related transcription factors into focus.

Genes specifically not responding to HL exposure in the double mutant, but responding in the wild type and the single mutants, might provide information on missing signals for induction or repression of these genes. At *t*_4h_ versus *t*_0_ only six HL-non-responsive genes were found in *adg1-1/tpt-2* (Supplementary Table S8B available at *JXB* online). In the wild type and both single mutants, the three up-regulated genes are non-characterized transporters (At4g13800 and At5g26220) or are involved in developmental processes (At1g77450). The three down-regulated genes are associated with ‘cell wall degradation’ (At1g02640), ‘redox’ (At3g62950; i.e. a putative glutaredoxin), and a trehalose phosphatase/synthase (TPS) 9 (At1g23870; Leyman *et al.*, 2001). A member of the same gene family (TPS1) is most probably involved in the initiation of flowering (Wahl *et al.*, 2013). There is, however, no direct link to the metabolic lesion in the mutant plant or to the putative signals involved. Likewise, the functional categories of genes not responding to HL in the double mutant are rather heterogeneous.

Further analyses of specifically regulated or non-regulated genes in the single mutant and the wild type are also presented in Supplementary Table S8 available at *JXB* online. Remarkably there were 87 genes specifically up-regulated in the *tpt-2* mutant 48h after LL/HL transfer. Of these genes, at least five genes were associated with Ca^2+^-dependent signalling and four with glutathione-*S*-transferases. In wild-type plants, the expression of only a low number of genes was specifically altered (Supplementary Table S8M, N available at *JXB* online).

### Are sugar-, phytohormone-, or oxidative stress-related signals involved in HL acclimation of *A. thaliana*?

The contribution of putative retrograde signals to global gene expression was assessed by comparing in-house with publicly available microarray data. Genes found to be HL responsive in the experimental set-up were compared with those genes regulated by elevated ‘sugar levels’, ‘oxidative stress’, or ‘hormones’ (for details, see Supplementary Tables S9 and S10 available at *JXB* online). Elevated sugar levels either were achieved by feeding *A. thaliana* seedlings with 3% Suc in light series (Michael *et al.*, 2008) or were obtained endogenously in the *pho3* mutant defective in SUT2, namely the Suc transporter responsible for phloem loading (Lloyd and Zakhleniuk, 2004). Oxidative stress based on singlet oxygen (^1^O_2_) and ^1^O_2_-dependent products from lipid or carotenoid oxidation (Shapiguzov *et al.*, 2013) can be observed in the *flu* mutant (op den Camp *et al.*, 2003). The FLU protein inhibits an early step in tetrapyrrole biosynthesis and thereby prevents the accumulation of the Chl precursor protochlorophyllide (Meskauskiene *et al.*, 2001). The application of the herbicide methyl viologen (MV) to *A. thaliana* leads to the accumulation of H_2_O_2_ (A. Ditzer, D. Bartels, and H.-H. Kirch; NASCARRAYS-143; op den Camp *et al.*, 2003). The effect of the phytohormones ABA, indole acetic acid (IAA), the cytokinin *trans*-zeatin (tZ), gibberellic acid (GA_3_), the ethylene precursor 1-aminocyclopropane-1-carboxylic acid (ACC), and methyl jasmonate (MJ) on global gene expression has been studied by Goda *et al.* (2008).

**Fig. 9.**
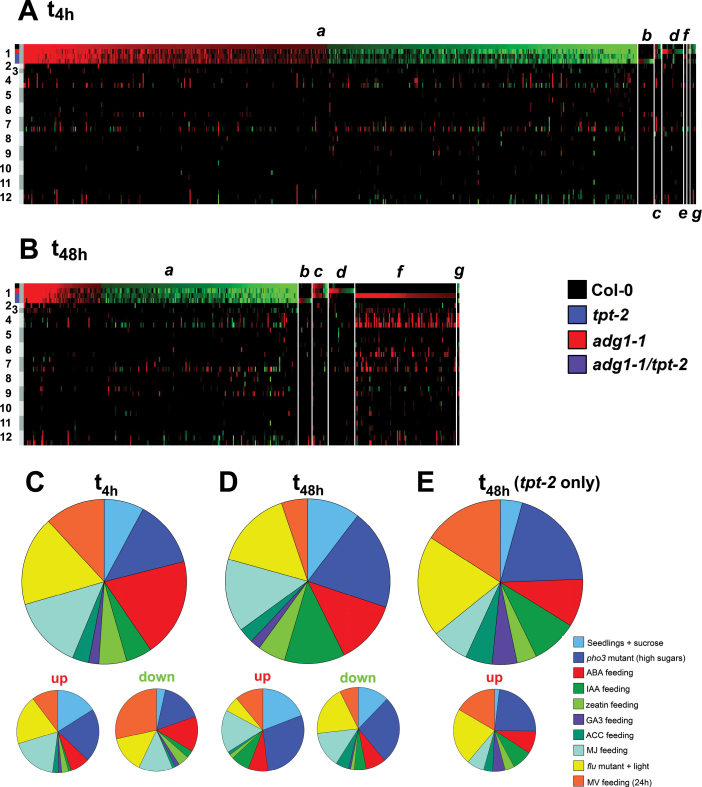
Heat map presentation of up- [red (log2_max_=3)] and down-regulated [green (log2_min_= –3)] genes of in-house microarrays at *t*_4h_ versus *t*_0_ (A) or *t*_48h_ versus *t*_0_ (B) compared with publicly available microarrays based on Supplementary Table S8 available at *JXB* online. The numbers to the left indicate in-house arrays (1) with Col-0, *tpt-2*, *adg1-1*, and *adg1-1/tpt-2* labelled with the colours black, blue, red, and dark purple, respectively; (2) seedlings+sucrose; (3) *pho3* mutant; (4) *flu* mutant; (5) *flu* mutant+MV; and feeding of the wild type with (6) MV, (7) ABA, (8) tZ, (9) IAA, (10) ACC, (11) GA_3_, or (12) MJ. The small italic letters indicate (a) commonly de-regulated genes in wild-type and all mutant plants of the in-house arrays, (b) specifically altered genes in *adg1-1/tpt-2*, (c) specifically not regulated genes in *adg1-1/tpt-2*, (d) specifically altered genes in *adg1-1*, (*e*) specifically not regulated genes in *adg1-1*, (f) specifically altered genes in *tpt-2*, and (g) specifically not regulated genes in *tpt-2*. Relative distribution of overlaps between differentially regulated genes in public microarrays compared with altered genes in all biotypes investigated in this study at *t*=4h (C) and *t*=48h (D) after LL/HL transfer. In (E), the relative distribution of overlaps between differentially regulated genes in publicly available microarrays is compared with up-regulated genes only found in the *tpt-2* mutant. The smaller pie charts refer to the relative distribution of up- or down-regulated genes between the individual experiments.

The major outcome of the microarray comparisons is summarized in Table 3, as well as in Fig. 9 and in Supplementary Fig. S3 available at *JXB* online. Genes responsive to external Suc feeding or enhanced endogenous carbohydrate levels in the *pho3* mutant represent a large portion of the commonly de-regulated genes shown in Fig. 9. Remarkably, the overlap of genes responsive to Suc feeding and in the *pho3* mutant was rather low (i.e. two out of 81 genes at *t*_4h_ and four out of 70 genes at *t*_48h_). Still, the direction of regulation (i.e. up or down) of HL- and sugar-responsive genes coincided to a high degree. More than 50% of the commonly up-regulated genes in the present experiments responded to sugars in the same way (Fig. 9). Furthermore, the log2 ratios of the individual data sets correlated directly with each other and resulted in Pearson correlation coefficients well above 0.7 (Table 3). There was no such coincidence or correlation with genes responding to phytohormones such as ABA (Fig. 9; Table 3). However, genes responding to MV-induced oxidative stress exhibited a positive long-term correlation (i.e. 48h after LL/HL transfer, Table 3), although the portion of ROS-induced genes was lower compared with *t*_4h_ versus *t*_0_ (Fig. 9). Interestingly, an entirely different approach undertaken by Oelze *et al.* (2012) used plants acclimated to extremely LL close to the light compensation point and their transfer to a 10- and 100-fold higher light intensity, respectively. Regulation of selected marker transcripts measured 6h after transfer to HL suggested that metabolite, redox, and lipid-derived signals are particularly important in realizing the acclimation.

Furthermore, the positive correlation with MJ is based on the fact that parts of the sugar-responsive genes also respond to MJ (e.g. 30% of the genes differentially regulated in *pho3*). It appears likely, therefore, that elevated sugar levels and ROS rather than phytohormones such as ABA are the predominant factors involved in the acclimation response in *A. thaliana*. However, the sugar-dependent response is obviously independent from the constraints in the mutant. Analyses of differentially regulated genes in the individual lines, including wild-type plants, revealed higher contributions of phytohormones or ROS to the HL response (Table 3; Supplementary Fig. S3 available at *JXB* online). To increase the number of genes for these comparisons, the gene lists from Supplementary Tables S5 and S6 available at *JXB* online [i.e. Venn areas (a), (b), (c), and (d) depicted in Fig. 7A and B; log2 ratio ±1.0] rather than from Supplementary Table S8 available at *JXB* online (log2 ratio ±1.5) were used.

Surprisingly, in the *tpt-2* single mutant, >25% of the highly up-regulated genes at *t*_48h_ and >30% of the moderately down-regulated genes also responded to ^1^O_2_ (Fig. 9E; Supplementary Fig. S3C available at *JXB* online). At *t*_4h_ there was a highly negative correlation between ROS- and HL-dependent gene expression in *tpt-2* (Table 3). This overall lower responsiveness towards ROS correlates well with aberrant levels of redox components (Asc and DHA) observed in this mutant (compare Fig. 4B). Genes individually up-regulated in *tpt-2* at *t*_48h_ after LL/HL transfer exhibited a good correlation with genes responsive to GA_3_. However, the total number of overlapping genes was low (eight genes). In wild-type plants, positive correlations of HL-responsive genes were found for sugars and MJ as well as for the phytohormone IAA, whereas tZ exhibited a negative correlation (Table 3). In the above cases as well as in the case of the negative correlation of HL- and sugar-responsive genes in the *adg1-1/tpt-2* double mutant, the portion of total genes available for these analyses was rather low (Supplementary Table S10 available at *JXB* online).

### Soluble sugars have a major impact on HL acclimation in *A. thaliana*

**Fig. 10.**
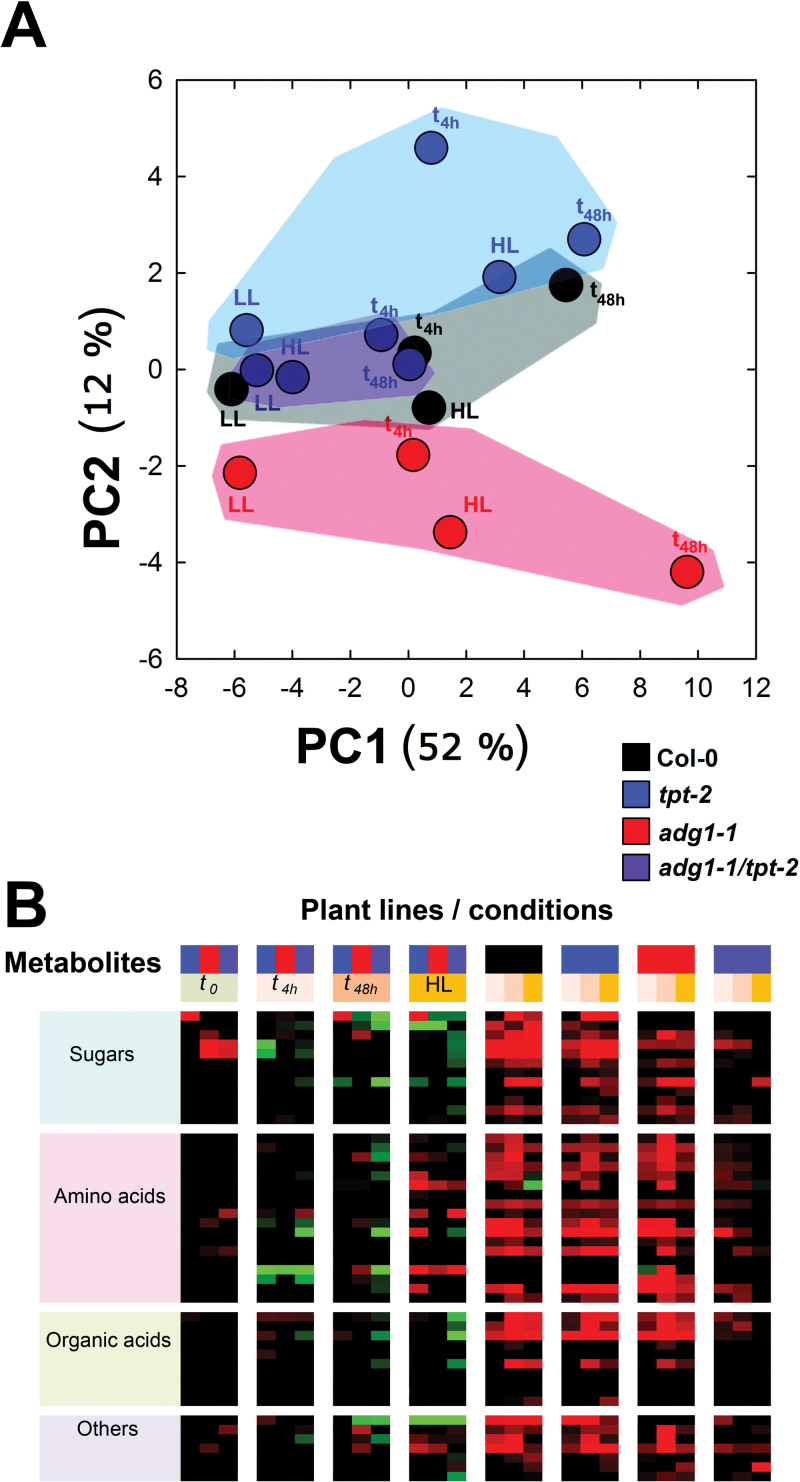
(A) Principle component analysis of metabolome data (see Supplementary Tables S11 and S12 available at *JXB* online) obtained with Col-0, *adg1-1*, *tpt-2*, and *adg1-1/tpt-2* grown in LL or HL and 4h (*t*_4h_) or 48h (*t*_48h_) after LL/HL transfer. (B) Heat map presentation of the same set of metabolome data shown in (A) referred either to Col-0 in a ‘static’ comparison or to *t*_0_ in a ‘dynamic’ comparison. The heatmap colours refer to increased or decreased metabolite contents at log2 ratios of +3 and –3 (bright red or bright green), respectively. Further colours represent Col-0 wild-type (black), *tpt-2* (blue), *adg1-1* (red), and *adg1-1/tpt-2* (dark purple). The different orange colour intensities are defined within the figure.

Metabolomics can help to identify retrograde signals (Caldana *et al.*, 2012). Profiles of 47 metabolites distributed amongst three major categories such as sugars (12), amino acids (18), organic acids (10), and others (seven) have been measured for all plants in this study. The plants were either grown continuously in LL or HL or were subjected to a LL/HL transfer and samples taken at *t*_4h_ and *t*_48h_. Figure 10 shows an overview of the metabolic profiling. More detailed data are contained in Table 4 and in Supplementary Tables S1, S11, and S12 available at *JXB* online. PCA (Fig. 10A) indicates that the first component of the metabolic profiles was separated based on the light conditions applied, whereas the second component was separated due to metabolic constraints in the respective mutants. Strikingly, for *adg1-1/tpt-2* there was neither a large separation in the first component nor was the second component clearly separated from the wild type, again showing that the double mutant is compromised in HL acclimation. The static and dynamic assessments of metabolite heat maps (Fig. 10B) indicate (i) that at *t*_0_ (i.e. in LL) the most pronounced increase exists for sugars, but not for amino acids or organic acids in *adg1-1* versus Col-0 and *adg1-1/tpt-2* versus Col-0. (ii) Upon transfer to HL, sugars and organic acids appear to be mostly decreased (most pronounced in *adg1-1/tpt-1* versus Col-0), whereas amino acids show a trend of only a transient decrease (at *t*_4h_ and *t*_48h_), but an increase at continuous growth in HL. (iii) As expected, most metabolites were elevated in Col-0 and both single mutants, at least transiently, in HL compared with LL. Strikingly, (iv) differences in metabolite contents are less marked in *adg1-1/tpt-2* grown in HL compared with LL conditions.

Table 4 shows that carbohydrate contents determined enzymatically or by GC-MC showed similar relative changes. Remarkably, Glc was the only soluble sugar that showed considerable variations in its content in *adg1-1/tpt-2*, in particular as a short-term response to LL/HL transfer (i.e. at *t*_4h_). In contrast to *adg1-1/tpt-2*, Glc contents remained high or further increased in wild-type and single mutant plants 48h after LL/HL transfer and when grown continuously in HL. It is hence tempting to speculate that a diminished Glc content in HL-grown *adg1-1/tpt-2* might contribute to the lack of HL acclimation of the double mutant, a notion that is further supported by the rescue of the double mutant’s HL phenotype by feeding of Glc (Heinrichs *et al.*, 2012; Schmitz *et al.*, 2012). Like Glc, Fru and Suc accumulated in wild-type and single mutant plants (at least transiently; Table 4A, 4B), whereas there was no such increase in *adg1-1/tpt-2.* It recently could be demonstrated that diminished cytosolic Fru contents due to the deficiency of SWEET17, an *A. thaliana* Fru exporter from the vacuole, resulted in plants with stunted growth (Chardon *et al.*, 2013). Hence, besides Glc, Fru might probably also be considered as a signalling molecule. Moreover, glycine, proline, and myo-inositol showed a similar distribution pattern in *adg1-1/tpt-2* to Glc. Proline (Verslues and Sharma, 2010) and myo-inositol (Donahue *et al.*, 2010) are involved in stress responses, and the latter is the structural basis for lipid signalling molecules (Valluru and van den Ende, 2011). Maltose contents were selectively increased in *tpt-2* starting 48h after LL/HL transfer, but were close to the limit of detection in the starch-free background. Maltose results from starch degradation via β-amylase and thus supports the idea of daytime starch mobilization in the absence of the TPT. The complete list of metabolites (Supplementary Table S11 available at *JXB* online) was further evaluated by a static and dynamic assessment, similar to the microarrays, statistically analysed (Table 5 of Supplementary Document S1 available at *JXB* online) and listed in Supplementary Table S12 available at *JXB* online.

## Conclusions

The genetic approach used here with mutants provides novel insight into the significance of carbohydrates and redox/ROS signalling in retrograde control of nuclear gene expression as a response of *A. thaliana* to HL exposure. Sugars are the first major products of photosynthesis and their contents are correlated with environmental conditions such as light and temperature. Even with an impaired day and night path of carbon export from the chloroplast in the *adg1-1/tpt-2* double mutant, some changes in Glc contents in response to HL were observed in the short term but not in the long term. In the group of commonly regulated genes, which respond to HL in the short term, a cross-talk of genes involved in carbohydrate and lipid metabolism exists. The impaired sugar-based long-term adaptation to HL observed in *adg1-1/tpt-2* most probably results in its severe growth and photosynthesis phenotype. To what extent severely down-regulated transcription factors such as MYC4 are involved in the lack of HL acclimation of *adg1-1/tpt-2* will be the subject of future experiments. Further elements, probably involved in the fine-tuning of the sugar response, have been identified amongst the genes which were either not or were specifically de-regulated in the single or double mutants. For instance, genes coding for heat shock proteins or genes that are involved in calcium signalling were specifically down-regulated in *adg1-1/tpt-2.* Detailed analyses of candidate genes would hence be challenging in future research. Meta-analyses of transcriptome data such as, for instance, conducted by van Akelen and Whelan (2012) can also shed more light on the fine-tuning in retrograde signalling. The meta-analyses conducted here support the idea that carbohydrates play a pivotal role in HL acclimation of *A. thaliana*.

## Supplementary data

Supplementary data are available at *JXB* online.

Figure S1. Correlation analyses of *Lhcb1* expression levels, ETR, and contents of MgProtoIX, soluble sugars, and total carbohydrates.

Figure S2. Graphviz presentation of co-expression networks obtained with ATTEDII for data shown in Supplementary Table S7A and Supplementary Document S3.

Figure S3. Relative distribution of differentially regulated genes in publicly available microarray experiments compared with in-house expression data for Col-0 (A), *tpt-2* (B), *adg1-1* (C), and *adg1-1/tpt-2* at *t*_4h_ and *t*_48h_ after LL/HL transfer.

Document S1. Statistical analyses (ANOVA/Tukey–Kramer) of the experimental data containing six tables.

Document S2. Detailed description of the static assessment of global gene expression data after LL/HL transfer between wild-type and mutant plants.

Document S3. Detailed analysis of commonly regulated genes following the dynamic assessment of global gene expression after LL/HL transfer between wild-type and mutant plants.

Table S1. Overview of the metabolite reporter list.

Table S2. Static comparison of global gene expression in *adg1-1*, *tpt-2*. and *adg1-1/tpt-2* versus Col-0 at *t*_0_ before LL/HL transfer.

Table S3. Static comparison of global gene expression in *adg1-1*, *tpt-2*, and *adg1-1/tpt-2* versus Col-0 at *t*_4h_ after LL/HL transfer.

Table S4. Static comparison of global gene expression in *adg1-1*, *tpt-2*, and *adg1-1/tpt-2* versus Col-0 at *t*_48h_ after LL/HL transfer.

Table S5. Dynamic comparison of global gene expression in *Col-0, adg1-1*, *tpt-2*, and *adg1-1/tpt-2* at *t*_4h_ after LL/HL transfer versus *t*_0_.

Table S6. Dynamic comparison of global gene expression in *Col-0, adg1-1*, *tpt-2*, and *adg1-1/tpt-2* at *t*_48h_ after LL/HL transfer versus *t*_0_.

Table S7. Filtered list of significantly (*P*<0.01) altered genes in the central overlapping regions of the Venn diagrams shown in Fig. 7A and B.

Table S8. List of genes specifically regulated or not regulated in the individual lines. The gene lists are based on the Venn diagrams shown in Fig. 7.

Table S9. Comparison of HL-responsive genes within the group of commonly regulated genes (Supplementary Table S7) with publicly available microarray data on genes responding to elevated sugars, oxidative stress, or the phytohormones ABA, indole acetic acid (IAA), *trans*-zeatin (tZ), gibberellic acid (GA_3_), the ethylene precursur ACC, and methyl jasmonate (MJ).

Table S10. Comparison of HL-responsive genes within the group of individually altered genes (Supplementary Table S8) with publicly available microarray data on genes responding to elevated sugars, oxidative stress, or the phytohormones ABA, indole acetic acid (IAA), *trans*-zeatin (tZ), gibberellic acid (GA_3_), the ethylene precursur ACC, and methyl jasmonate (MJ).

Table S11. Contents of metabolites determined by GC-MS in leaves of (A) Col-0, (B) *adg1-1*, (C) *tpt-2*, and (D) *adg1-1/tpt-2* grown either continuously in LL or HL or after transfer from LL to HL at *t*_4h_ or *t*_48h_.

Table S12. Evaluation of metabolite profiles in response to growth in LL or HL or after LL/HL transfer in Col-0 wild-type, *adg1-1*, and *tpt-2* single mutants as well as *adg1-1/tpt-2* double mutant plants based on the data in Supplementary Table S10.

Supplementary Data
