# Supplementary material for: The essential role of sugar metabolism in the acclimation response of Arabidopsis thaliana to high light intensities
Source: J Exp Bot. 2014 Feb 12;65(6):1619–36. doi: 10.1093/jxb/eru027 (PMC3967092; doi:10.1093/jxb/eru027)
Supplement: Supplementary Data [file supp_65_6_1619__index.html]

The essential role of sugar metabolism in the acclimation response of Arabidopsis thaliana to high light intensities — The essential role of sugar metabolism in the acclimation response of Arabidopsis thaliana to high light intensities — Supplementary Data 

# The essential role of sugar metabolism in the acclimation response of *Arabidopsis thaliana* to high light intensities

## Supplementary Data

Data files

**Files in this Data Supplement:**

- Supplementary Data - Supplementary Data
- Supplementary Data - Supplementary Data
- Supplementary Data - Supplementary Data
- Supplementary Data - Supplementary Data
- Supplementary Data - Supplementary Data
- Supplementary Data - Supplementary Data
- Supplementary Data - Supplementary Data
- Supplementary Data - Supplementary Data
- Supplementary Data - Supplementary Data
- Supplementary Data - Supplementary Data
- Supplementary Data - Supplementary Data
- Supplementary Data - Supplementary Data
