# Supplementary material for: The essential role of sugar metabolism in the acclimation response of Arabidopsis thaliana to high light intensities
Source: J Exp Bot. 2014 Feb 12;65(6):1619–36. doi: 10.1093/jxb/eru027 (PMC3967092; doi:10.1093/jxb/eru027)
Supplement: Supplementary Data [file supp_eru027_jexbot114363_file001.pdf]

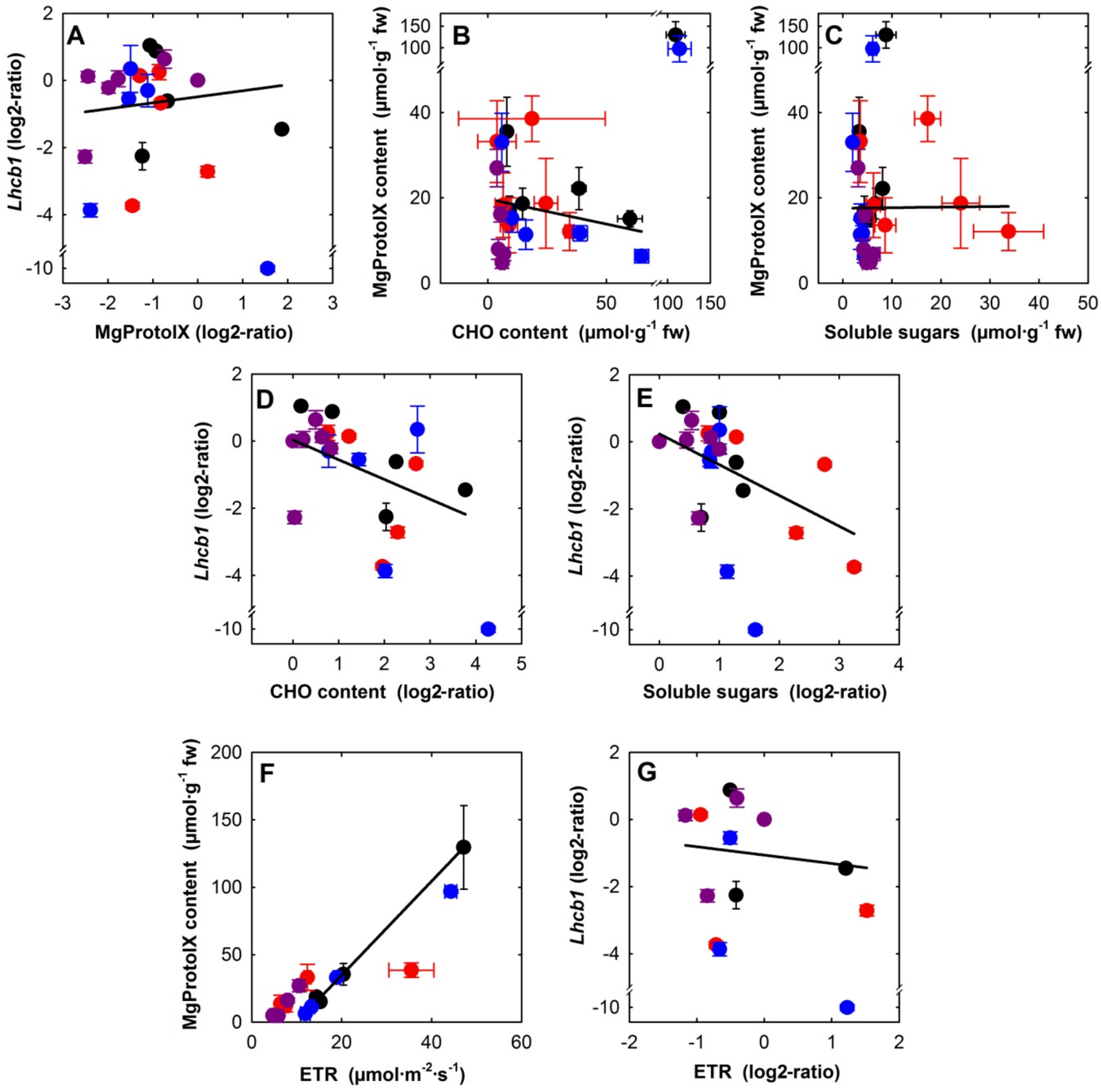

Supplementary Fig. S1. Correlation analyses of *Lhcb1* expression levels, ETR, as well as contents of MgProtoIX, soluble sugars and total carbohydrates. (**A**) *Lhcb1* expression *vs* MgProtoIX contents. MgProtoIX *vs* total carbohydrates (**B**), soluble sugars (**C**), or ETR (**F**). *Lhcb1* expression *vs s* total carbohydrates (**D**), soluble sugars (**E**), or ETR (**G**). The data were obtained from Figs. 1 to 3. Where necessary log2-ratios instead of absolute values were used. The colours represent Col-0 (black), *tpt-2* (blue), *adg1-1* (red) and *adg1-1/tpt-2* (dark purple).

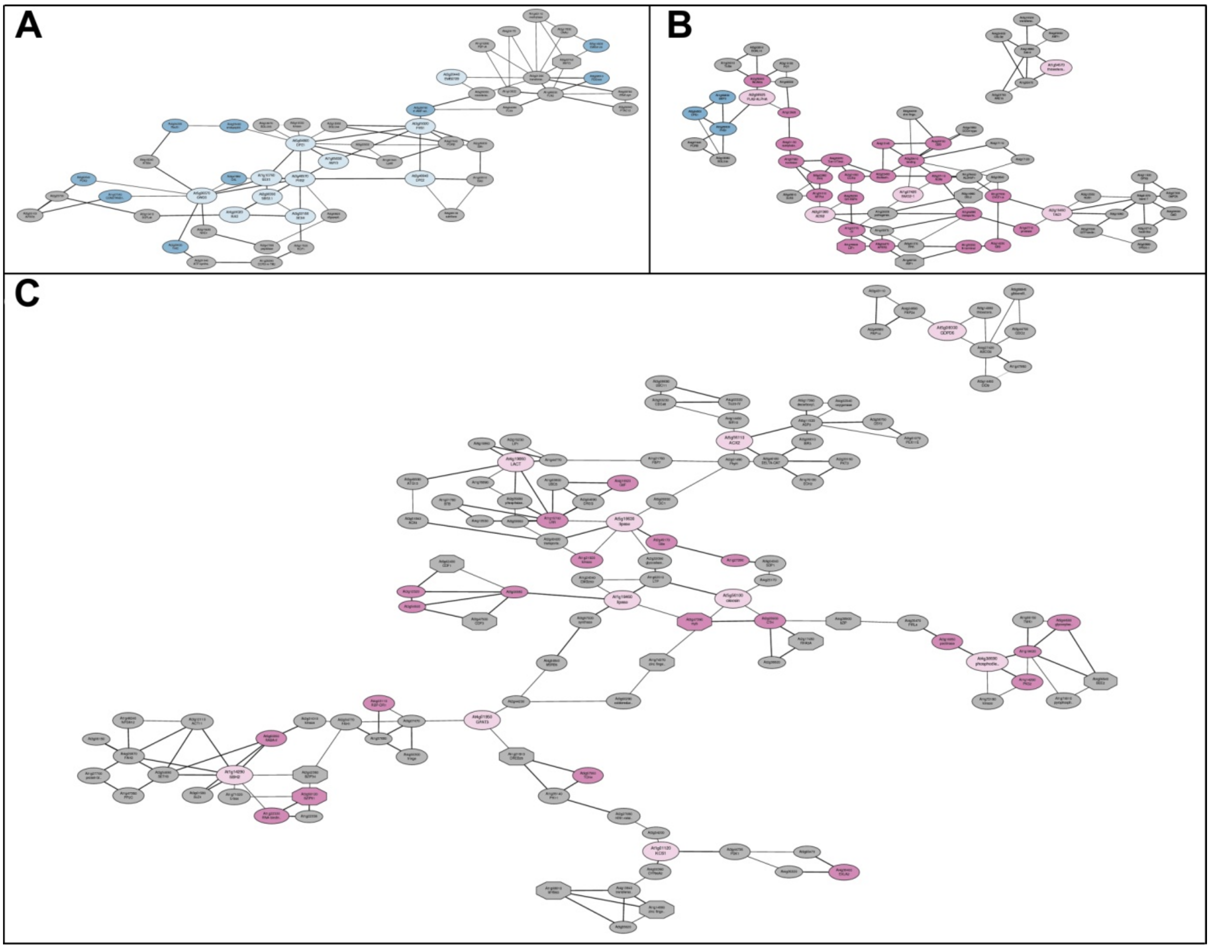

Supplementary Fig. S2. Graphviz presentation of co-expression networks obtained with ATTEDII for data shown in Supplementary Table S7A and Supplementarty Document S3. The query genes were found amongst the commonly regulated genes 4h after LL/HL-transfer. `Query genes´ related to major CHO metabolism (up-regulated) (**A**), lipid metabolism up- (**B**) or down-regulated (**C**), are marked in light blue or purple colour, respectively. Co-expressed genes indentified as differentially regulated in the array experiment are marked in dark-blue or purple colour. All other genes within the co-expression network are marked in grey. The dark blue-coloured genes marked in (**B**) are associated with major CHO metabolism and were co-expressed with `query genes´ for lipid metabolism.

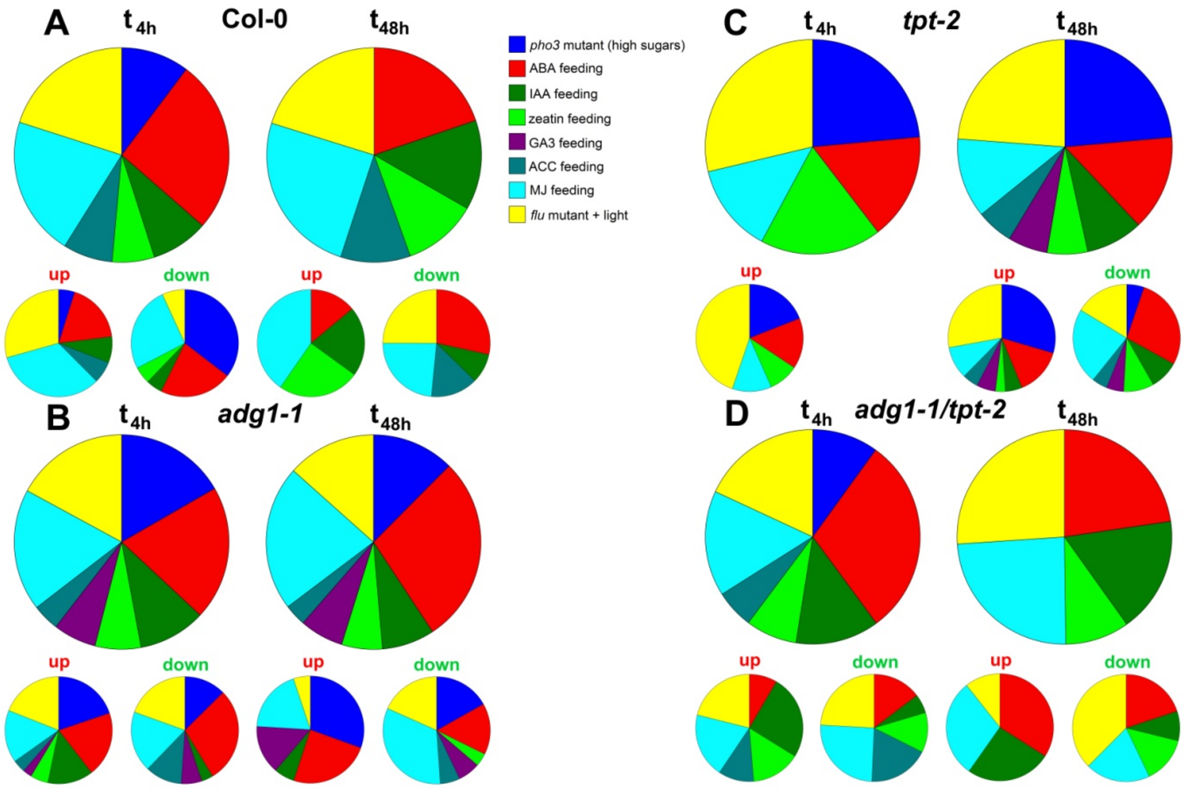

Supplementary Figure S3. Relative distribution of differentially regulated genes in publicly available microarray experiments compared to in-house expression data for Col-0 (**A**), *tpt-2* (**B**), *adg1-1* (**C**) and *adg1-1/tpt-2* at t_{4h} and t_{48h} after LL/HL-transfer.

Supplementary Table S11. Contents of metabolites determined by GC/MS in leaves of **(A)** Col-0, **(B)** *adg1-1*, **(C)** *tpt-2* and **(D)** *adg1-1/tpt-2* grown either continuously in LL or HL or after a LL/HL-transfer at t_{4h} or t_{48h}. The data represent the mean of five independent samples ± SE.

A

**Metabolites**	**Relative metabolite content (arbitrary units·g^{-1} fw)**			
**Sugars**	**Col-0 t_0 (LL)**	**Col-0 t_{4h}**	**Col-0 t_{48h}**	**Col-0 (HL)**
D-Sucrose	3.255 ± 0.062	6.083 ± 0.101	7.325 ± 0.124	6.776 ± 0.190
D-Glucose	0.532 ± 0.039	21.348 ± 0.680	12.206 ± 1.136	13.711± 1.251
D-Fructose	0.737 ± 0.042	16.803 ± 0.449	9.683 ± 1.846	12.775 ± 0.897
D-Mannose	0.146 ± 0.012	0.496 ± 0.044	0.967 ± 0.572	1.215 ± 0.104
D-Maltose	0.122 ± 0.007	0.250 ± 0.020	1.871 ± 0.255	0.519 ± 0.027
α,α'- D-Trehalose	0.247 ± 0.026	0.476 ± 0.030	4.097 ± 0.431	2.844 ± 0.169
Raffinose	0.073 ± 0.015	0.369 ± 0.061	0.197 ± 0.027	2.117 ± 0.527
1,6-anhydro, β-D-Glucose	6.101 ± 0.939	8.124 ± 1.125	14.672 ± 1.616	10.773 ± 1.493
1-O-methyl-, α-D-Mannopyranoside	3.525 ± 0.158	3.642 ± 0.375	9.498 ± 0.492	6.665 ± 0.309
DL-Fucose	0.815 ± 0.025	2.280 ± 0.199	3.359 ± 0.314	3.463 ± 0.180
D-Arabinose	0.852 ± 0.054	0.609 ± 0.026	1.350 ± 0.066	1.502 ± 0.113
Glucoheptose	0.105 ± 0.012	0.331 ± 0.031	0.518 ± 0.062	0.400 ± 0.011
**Amino acids**	**Col-0 t_0 (LL)**	**Col-0 t_{4h}**	**Col-0 t_{48h}**	**Col-0 (HL)**
DL-Glutamic acid	32.455 ± 1.864	124.727 ± 2.566	165.011 ± 2.397	39.409 ± 3.015
L-Aspartic acid	9.675 ± 0.164	9.138 ± 0.408	18.602 ± 1.704	6.243 ± 0.738
DL-Asparagine	n.d.	0.439 ± 0.167	3.457 ± 1.799	0.063 ± 0.007
DL-Alanine	1.700 ± 0.084	3.270 ± 0.615	5.075 ± 1.160	0.286 ± 0.039
Glycine	3.515 ± 0.228	144.514 ± 2.407	115.600 ± 1.315	14.483 ± 0.327
DL-Serine	10.116 ± 0.358	28.045 ± 0.745	54.533 ± 1.411	38.380 ± 0.840
DL-Threonine	12.512 ± 0.546	19.471 ± 0.774	31.469 ± 0.879	10.606 ±0.532
DL-Cysteine	0.611 ± 0.041	1.021 ± 0.138	2.313 ± 0.511	1.789 ± 0.212
DL-Methionine	0.168 ± 0.012	0.852 ± 0.077	0.823 ± 0.174	0.069 ± 0.014
L-Isoleucine	0.803 ± 0.014	3.146 ± 0.190	3.806 ± 0.259	3.342 ± 0.077
DL-Valine	3.195 ± 0.098	14.803 ± 0.463	16.162 ± 0.968	10.188 ± 0.458
L-Lysine	0.195 ± 0.009	0.509 ± 0.037	0.649 ± 0.082	0.361 ± 0.031
DL-Arginine, -NH3	0.082 ± 0.013	0.339 ± 0.022	0.826 ± 0.394	0.094 ± 0.008
DL-Phenylalanine	0.723 ± 0.042	9.609 ± 0.630	5.678 ± 1.749	2.007 ± 0.095
DL-Tyrosine	n.d.	0.430 ± 0.042	0.443 ± 0.123	0.151 ± 0.020
L-Tryptophan	0.440 ± 0.016	0.662 ± 0.052	0.778 ± 0.038	0.722 ± 0.027
L-Proline	7.164 ± 0.833	35.885 ± 1.733	60.061 ± 3.783	14.332 ± 2.103
β-Alanine	0.159 ± 0.011	0.785 ± 0.065	1.383 ± 0.121	0.704 ± 0.051

A (continued)

Organic acids	Col-0 t_0 (LL)	Col-0 t_{4h}	Col-0 t_{48h}	Col-0 (HL)
Pyruvic acid	0.610 ± 0.034	0.667 ± 0.061	0.967 ± 0.067	0.841 ± 0.048
2-methyl-DL-Malic acid	0.094 ± 0.007	0.338 ± 0.029	1.023 ± 0.028	0.516 ± 0.025
Glutaric acid	0.846 ± 0.058	1.611 ± 0.235	5.958 ± 0.397	4.389 ± 0.098
Succinic acid	0.667 ± 0.029	1.350 ± 0.114	6.427 ± 0.975	3.803 ± 0.257
Fumaric acid	106.355 ± 3.898	125.302 ± 1.852	111.554 ± 2.943	133.314 ± 2.247
Malonic acid	n.d.	0.197 ± 0.022	0.193 ± 0.022	0.124 ± 0.018
DL-Glyceric acid	0.581 ± 0.016	2.972 ± 0.359	3.602 ± 0.100	6.238 ± 0.211
3-hydroxy-Butyric acid	0.299 ± 0.017	0.399 ± 0.051	0.733 ± 0.069	0.436 ± 0.025
D-Gluconic acid or D-Galactonic acid	7.233 ± 0.486	11.108 ± 1.195	15.170 ± 1.927	20.526 ± 0.506
Benzoic acid	5.339 ± 0.149	6.958 ± 0.712	7.477 ± 0.391	6.589 ± 0.154

Miscellaneous	Col-0 t_0 (LL)	Col-0 t_{4h}	Col-0 t_{48h}	Col-0 (HL)
Glycerol	1.502 ± 0.067	2.968 ± 0.304	2.913 ± 0.140	3.985 ± 0.468
myo-Inositol	12.818 ± 0.580	19.915 ± 0.402	23.725 ± 1.369	26.920 ± 0.374
Galactinol	0.091 ± 0.012	0.571 ± 0.062	0.970 ± 0.174	4.876 ± 0.910
Erythritol	0.272 ± 0.012	0.730 ± 0.088	1.060 ± 0.073	0.821 ± 0.049
Tyramine	0.408 ± 0.013	0.797 ± 0.073	0.991 ± 0.154	1.452 ± 0.036
DL-Ornithine	0.601 ± 0.021	2.979 ± 0.132	5.020 ± 1.605	0.569 ± 0.098
Putrescine	1.086 ± 0.073	7.529 ± 0.641	10.936 ± 0.941	3.108 ± 0.486

B

**Metabolites**	**Relative metabolite content (arbitrary units·g^{-1} fw)**			
**Sugars**	***adg1-1*** **t_0 (LL)**	***adg1-1*** **t_{4h}**	***adg1-1*** **t_{48h}**	***adg1-1*** **(HL)**
D-Sucrose	5.662 ± 0.184	6.691 ± 0.061	7.080 ± 0.047	7.897 ± 0.118
D-Glucose	6.765 ± 0.528	25.970 ± 0.174	24.411 ± 0.440	26.822 ± 0.368
D-Fructose	10.905 ± 0.632	20.173 ± 0.213	19.711 ± 0.366	20.947 ± 0.336
D-Mannose	0.486 ± 0.017	1.116 ± 0.115	3.837 ± 0.181	1.859 ± 0.126
D-Maltose	n.d.	0.545 ± 0.030	0.517 ± 0.044	0.142 ± 0.012
α,α'- D-Trehalose	0.162 ± 0.013	0.437 ± 0.034	3.334 ± 0.229	1.476 ± 0.066
Raffinose	n.d.	0.168 ± 0.019	0.074 ± 0.007	0.111 ± 0.015
1,6-anhydro, β-D-Glucose	7.350 ± 0.730	16.062 ± 0.622	18.570 ± 1.735	22.615 ± 3.391
1-O-methyl-, α-D-Mannopyranoside	3.547 ± 0.333	1.722 ± 0.139	1.457 ± 0.031	2.071 ± 0.223
DL-Fucose	1.303 ± 0.034	2.939 ± 0.289	4.932 ± 0.199	4.252 ± 0.280
D-Arabinose	1.070 ± 0.032	0.654 ± 0.043	2.080 ± 0.205	2.795 ± 2.265
Glucoheptose	0.167 ± 0.010	0.444 ± 0.018	0.781 ± 0.033	0.529 ± 0.035
**Amino acids**	***adg1-1*** **t_0 (LL)**	***adg1-1*** **t_{4h}**	***adg1-1*** **t_{48h}**	***adg1-1*** **(HL)**
DL-Glutamic acid	29.829 ± 1.808	65.857 ± 5.493	176.494 ± 1.888	37.772 ± 1.714
L-Aspartic acid	7.341 ± 0.280	8.407 ± 0.426	13.491 ± 0.952	5.025 ± 0.400
DL-Asparagine	0.193 ± 0.056	0.062 ± 0.013	12.021 ± 1.145	0.273 ± 0.065
DL-Alanine	1.778 ± 0.095	2.735 ± 0.452	10.569 ± 1.408	1.272 ± 0.161
Glycine	4.776 ± 0.235	84.629 ± 3.231	116.061 ± 1.686	22.941 ± 1.590
DL-Serine	6.097 ± 0.222	23.157 ± 1.375	49.678 ± 1.476	25.879 ± 1.761
DL-Threonine	16.884 ± 0.636	21.588 ± 1.051	33.180 ± 1.800	20.370 ± 1.934
DL-Cysteine	0.941 ± 0.035	1.374 ± 0.136	4.577 ± 0.420	3.200 ± 0.250
DL-Methionine	0.137 ± 0.016	0.485 ± 0.058	0.532 ± 0.066	0.106 ± 0.011
L-Isoleucine	0.925 ± 0.025	2.984 ± 0.338	3.552 ± 0.203	2.962 ± 0.256
DL-Valine	3.308 ± 0.092	12.348 ± 0.903	19.391 ± 1.093	12.170 ± 0.954
L-Lysine	0.284 ± 0.011	0.635 ± 0.038	0.961 ± 0.082	0.534 ± 0.049
DL-Arginine, -NH3	0.074 ± 0.007	0.335 ± 0.037	2.837 ± 0.324	0.221 ± 0.023
DL-Phenylalanine	1.869 ± 0.039	12.419 ± 1.041	14.334 ± 0.922	3.100 ± 0.172
DL-Tyrosine	0.060 ± 0.003	0.428 ± 0.031	1.004 ± 0.085	0.250 ± 0.040
L-Tryptophan	0.334 ± 0.016	0.564 ± 0.018	0.638 ± 0.018	0.581 ± 0.029
L-Proline	4.014 ± 0.293	30.162 ± 2.257	67.997 ± 7.015	18.805 ± 2.639
β-Alanine	0.353 ± 0.022	0.585 ± 0.078	2.124 ± 0.108	0.855 ± 0.057

B (continued)

Organic acids	*adg1-1* t_0 (LL)	*adg1-1* t_{4h}	*adg1-1* t_{48h}	*adg1-1* (HL)
Pyruvic acid	0.527 ± 0.043	0.525 ± 0.043	0.870 ± 0.050	1.086 ± 0.061
2-Methyl-DL-malic acid	0.121 ± 0.009	0.357 ± 0.033	1.132 ± 0.064	0.616 ± 0.023
Glutaric acid	1.075 ± 0.026	1.827 ± 0.183	5.075 ± 0.333	3.033 ± 0.141
Succinic acid	1.045 ± 0.040	3.746 ± 0.427	13.658 ± 0.547	4.391 ± 0.164
Fumaric acid	136.964 ± 4.410	115.525 ± 1.695	98.817 ± 3.491	116.770 ± 2.312
Malonic acid	n.d.	0.105 ± 0.018	0.176 ± 0.034	0.069 ± 0.012
DL-Glyceric acid	0.395 ± 0.008	2.993 ± 0.358	4.656 ± 0.256	3.977 ± 0.216
3-hydroxy-Butyric acid	0.210 ± 0.015	0.335 ± 0.039	0.589 ± 0.067	0.331 ± 0.051
D-Gluconic acid or D-Galactonic acid	10.026 ± 0.467	12.481 ± 1.317	14.139 ± 2.164	14.509 ± 1.642
Benzoic acid	4.206 ± 0.339	6.773 ± 0.685	6.524 ± 0.305	7.034 ± 0.272
**Miscellaneous**	*adg1-1* t_0 (LL)	*adg1-1* t_{4h}	*adg1-1* t_{48h}	*adg1-1* (HL)
Glycerol	1.243 ± 0.024	2.197 ± 0.245	2.552 ± 0.047	4.424 ± 1.145
myo-Inositol	6.351 ± 0.345	13.788 ± 0.778	18.833 ± 0.741	16.165 ± 0.538
Galactinol	n.d.	0.310 ± 0.034	0.170 ± 0.015	0.473 ± 0.077
Tyramine	0.444 ± 0.020	0.938 ± 0.084	0.643 ± 0.111	0.877 ± 0.086
Erythritol	0.527 ± 0.039	1.070 ± 0.125	4.685 ± 0.379	1.436 ± 0.093
DL-Ornithine	1.059 ± 0.047	2.072 ± 0.244	11.812 ± 1.380	1.245 ± 0.135
Putrescine	2.931 ± 0.101	9.603 ± 1.257	36.145 ± 2.688	11.367 ± 1.118

C

Metabolites	Relative metabolite content (arbitrary units·g^{-1} fw)			
**Sugars**	***tpt-2*** **t_0 (LL)**	***tpt-2*** **t_{4h}**	***tpt-2*** **t_{48h}**	***tpt-2*** **(HL)**
D-Sucrose	3.386 ± 0.063	4.175 ± 0.157	7.152 ± 0.127	8.134 ± 0.248
D-Glucose	0.666 ± 0.044	3.189 ± 0.224	22.965 ± 0.685	16.006 ± 0.748
D-Fructose	0.913 ± 0.032	3.376 ± 0.236	17.424 ± 0.464	8.609 ± 0.564
D-Mannose	0.191 ± 0.017	0.462 ± 0.041	0.769 ± 0.030	0.562 ± 0.059
D-Maltose	0.886 ± 0.045	0.509 ± 0.055	10.976 ± 0.813	6.708 ± 0.954
α,α'- D-Trehalose	0.248 ± 0.016	0.314 ± 0.029	1.223 ± 0.126	0.838 ± 0.080
Raffinose	0.104 ± 0.010	0.350 ± 0.057	0.250 ± 0.022	0.139 ± 0.023
1,6-anhydro, β-D-Glucose	3.593 ± 0.493	9.476 ± 1.402	10.553 ± 0.445	14.375 ± 2.019
1-O-methyl-, α-D-Mannopyranoside	6.533 ± 0.223	7.662 ± 0.506	2.200 ± 0.164	5.778 ± 0.810
DL-Fucose	0.920 ± 0.054	1.963 ± 0.130	3.657 ± 0.173	2.427 ± 0.341
D-Arabinose	0.815 ± 0.096	0.723 ± 0.060	1.221 ± 0.092	2.577 ± 0.249
Glucoheptose	0.096 ± 0.022	0.325 ± 0.043	0.374 ± 0.056	0.222 ± 0.021
**Amino acids**	***tpt-2*** **t_0 (LL)**	***tpt-2*** **t_{4h}**	***tpt-2*** **t_{48h}**	***tpt-2*** **(HL)**
DL-Glutamic acid	53.294 ± 2.592	98.735 ± 6.497	169.430 ± 3.908	87.257 ± 9.739
L-Aspartic acid	12.935 ± 0.680	18.301 ± 1.220	19.163 ± 1.581	13.648 ± 1.869
DL-Asparagine	n.d.	0.045 ± 0.009	2.847 ± 0.382	0.820 ± 0.381
DL-Alanine	2.357 ± 0.057	3.526 ± 0.258	10.505 ± 0.598	3.275 ± 0.229
Glycine	4.407 ± 0.258	107.478 ± 1.701	109.623 ± 2.306	80.016 ± 3.368
DL-Serine	15.969 ± 0.706	65.008 ± 0.910	66.137 ± 1.239	63.031 ± 1.454
DL-Threonine	15.830 ± 0.973	42.280 ± 1.347	39.311 ± 1.212	44.857 ± 1.726
DL-Cysteine	0.558 ± 0.064	1.255 ± 0.155	2.019 ± 0.116	2.007 ± 0.316
DL-Methionine	0.238 ± 0.023	1.074 ± 0.155	0.782 ± 0.117	0.328 ± 0.061
L-Isoleucine	1.038 ± 0.041	3.647 ± 0.383	4.447 ± 0.150	3.851 ± 0.326
DL-Valine	4.621 ± 0.156	15.562 ± 1.432	24.076 ± 1.064	17.124 ± 1.605
L-Lysine	0.242 ± 0.017	0.488 ± 0.071	0.617 ± 0.033	0.637 ± 0.063
DL-Arginine, -NH3	0.128 ± 0.019	0.256 ± 0.039	0.677 ± 0.032	0.286 ± 0.055
DL-Phenylalanine	0.911 ± 0.037	3.588 ± 0.331	5.816 ± 0.289	3.486 ± 0.489
DL-Tyrosine	n.d.	0.102 ± 0.024	0.493 ± 0.021	0.229 ± 0.028
L-Tryptophan	0.492 ± 0.030	0.644 ± 0.044	0.731 ± 0.029	0.554 ± 0.033
L-Proline	6.839 ± 0.425	70.172 ± 4.469	42.499 ± 4.535	70.521 ± 5.587
β-Alanine	0.187 ± 0.008	0.862 ± 0.083	1.800 ± 0.092	1.360 ± 0.134

C (continued)

**Organic acids**	***tpt-2* t_0 (LL)**	***tpt-2* t_{4h}**	***tpt-2* t_{48h}**	***tpt-2* (HL)**
Pyruvic acid	0.794 ± 0.059	1.637 ± 0.176	1.069 ± 0.037	1.612 ± 0.190
2-Methyl-DL-malic acid	0.166 ± 0.007	0.416 ± 0.017	1.213 ± 0.043	0.557 ±0.050
Glutaric acid	0.947 ± 0.045	1.632 ± 0.126	5.024 ± 0.269	2.771 ± 0.353
Succinic acid	1.447 ± 0.058	3.816 ± 0.270	10.514 ± 0.443	8.221 ± 0.919
Fumaric acid	117.778 ± 3.673	101.630 ± 2.485	91.506 ± 2.352	112.648 ± 3.501
Malonic acid	n.d.	0.207 ± 0.020	0.204 ± 0.015	0.247 ± 0.153
DL-Glyceric acid	0.804 ± 0.038	7.920 ± 0.485	8.878 ± 0.388	7.281 ± 0.698
3-hydroxy-Butyric acid	0.408 ± 0.018	0.560 ± 0.037	0.794 ± 0.042	0.524 ± 0.069
D-Gluconic acid or D-Galactonic acid	7.160 ± 0.460	11.984 ± 1.322	14.063 ± 1.256	25.587 ±3.280
Benzoic acid	5.736 ± 0.143	7.487 ± 0.184	7.347 ± 0.295	8.712 ± 0.887

**Miscellaneous**	***tpt-2* t_0 (LL)**	***tpt-2* t_4**	***tpt-2* t_{48}**	***tpt-2* (HL)**
Glycerol	1.415 ± 0.048	2.790 ± 0.236	2.413 ± 0.127	3.547 ± 0.182
myo-Inositol	11.443 ± 0.722	18.019 ± 0.436	23.179 ± 0.533	15.782 ± 0.559
Galactinol	0.127 ± 0.013	1.549 ± 0.204	1.312 ± 0.123	0.413 ± 0.047
Tyramine	0.422 ± 0.032	0.679 ± 0.048	0.854 ± 0.086	1.120 ± 0.134
Erythritol	0.352 ± 0.031	0.779 ± 0.062	1.283 ± 0.067	1.287 ± 0.161
DL-Ornithine	0.708 ± 0.047	1.835 ± 0.304	4.192 ± 0.348	1.745 ± 0.218
Putrescine	1.140 ± 0.054	3.902 ± 0.345	16.551 ± 0.999	14.764 ± 1.628

D

**Metabolites**	**Relative metabolite content (arbitrary units·g^{-1} fw)**			
**Sugars**	***adg1-1/tpt-2* t_0 (LL)**	***adg1-1/tpt-2* t_{4h}**	***adg1-1/tpt-2* t_{48h}**	***adg1-1/tpt-2* (HL)**
D-Sucrose	3.790 ± 0.149	5.294 ± 0.073	5.928 ± 0.082	4.770 ± 0.186
D-Glucose	2.923 ± 0.439	13.410 ± 1.300	10.768± 0.736	5.729 ± 0.771
D-Fructose	3.924 ± 0.476	5.836 ± 0.473	8.440 ± 0.537	3.758 ± 0.287
D-Mannose	0.300 ± 0.017	0.737 ± 0.085	0.582 ± 0.012	0.353 ± 0.022
D-Maltose	n.d.	0.379 ± 0.043	0.205 ± 0.017	0.141 ± 0.034
α,α'- D-Trehalose	0.152 ± 0.019	0.178 ± 0.019	0.250 ± 0.026	0.759 ± 0.098
Raffinose	n.d.	0.137 ± 0.016	0.032 ± 0.003	n.d.
1,6-anhydro, β-D-Glucose	5.285 ± 1.062	14.692 ± 1.139	16.991 ± 1.032	6.848 ± 1.479
1-O-methyl-, α-D-Mannopyranoside	4.754 ± 0.185	4.072 ± 0.258	5.782 ± 0.318	3.755 ± 0.359
DL-Fucose	1.145 ± 0.042	2.504 ± 0.330	1.623 ± 0.038	1.152 ± 0.102
D-Arabinose	1.283 ± 0.081	0.891 ± 0.104	1.871 ± 0.101	1.404 ± 0.109
Glucoheptose	0.152 ± 0.014	0.314 ± 0.042	0.378 ± 0.019	0.137 ± 0.010
**Amino acids**	***adg1-1/tpt-2* t_0 (LL)**	***adg1-1/tpt-2* t_{4h}**	***adg1-1/tpt-2* t_{48h}**	***adg1-1/tpt-2* (HL)**
DL-Glutamic acid	35.303 ± 1.804	73.146 ± 5.631	70.013 ± 4.776	37.772 ± 4.500
L-Aspartic acid	10.986 ± 0.890	13.559 ± 0.590	15.069 ± 1.222	11.318 ± 1.376
DL-Asparagine	n.d.	0.030 ± 0.003	0.125 ± 0.032	0.505 ± 0.174
DL-Alanine	2.148 ± 0.188	5.947 ± 0.495	6.390 ± 0.614	0.950 ± 0.080
Glycine	2.523 ± 0.148	20.289 ± 1.631	7.615 ± 0.377	4.302 ± 0.255
DL-Serine	7.704 ± 0.517	19.015 ± 1.191	16.563 ± 0.494	14.933 ± 0.676
DL-Threonine	49.848 ± 0.911	68.305 ± 4.333	51.722 ± 2.373	72.887 ± 4.955
DL-Cysteine	0.791 ± 0.105	1.359 ± 0.183	2.691 ± 0.209	1.060 ± 0.156
DL-Methionine	0.125 ± 0.029	0.387 ± 0.070	0.269 ± 0.037	0.118 ± 0.018
L-Isoleucine	1.233 ± 0.052	3.347 ± 0.234	3.065 ± 0.178	2.165 ± 0.114
DL-Valine	4.867 ± 0.303	13.861 ± 0.900	12.719 ± 0.614	6.813 ± 0.446
L-Lysine	0.369 ± 0.019	0.604 ± 0.086	0.657 ± 0.029	0.523 ± 0.052
DL-Arginine, -NH3	0.092 ± 0.011	0.219 ± 0.026	0.194 ± 0.011	0.097 ± 0.010
DL-Phenylalanine	1.341 ± 0.115	3.051 ± 0.259	2.354 ± 0.112	1.095 ± 0.104
DL-Tyrosine	n.d.	0.092 ± 0.027	0.167 ± 0.018	0.121 ± 0.021
L-Tryptophan	0.482 ± 0.015	0.561 ± 0.041	0.632 ± 0.009	0.514 ± 0.037
L-Proline	6.943 ± 1.310	26.598 ± 1.854	22.410 ± 4.796	9.856 ± 1.323
β-Alanine	0.435 ± 0.016	0.614 ± 0.048	1.475 ± 0.046	1.003 ± 0.073

D (continued)

Organic acids	*adg1-1/tpt-2* t_0 (LL)	*adg1-1/tpt-2* t_{4h}	*adg1-1/tpt-2* t_{48h}	*adg1-1/tpt-2* (HL)
Pyruvic acid	0.623 ± 0.032	1.098 ± 0.066	1.085 ± 0.119	0.624 ± 0.024
2-Methyl-DL-malic acid	0.163 ± 0.017	0.380 ± 0.013	0.615 ± 0.021	0.164 ± 0.019
Glutaric acid	1.089 ± 0.039	1.516 ± 0.179	2.077 ± 0.054	1.113 ± 0.051
Succinic acid	1.055 ± 0.070	3.552 ± 0.184	2.543 ± 0.075	0.707 ± 0.050
Fumaric acid	96.776 ± 1.606	91.044 ± 1.664	85.786 ± 1.356	82.427 ± 10.166
Malonic acid	n.d.	0.141 ± 0.022	0.141 ± 0.008	0.077 ± 0.008
DL-Glyceric acid	0.401 ± 0.012	0.957 ± 0.103	0.897 ± 0.038	0.784 ± 0.056
3-hydroxy-Butyric acid	0.381 ± 0.024	0.502 ± 0.056	0.513 ± 0.020	0.308 ± 0.044
D-Gluconic acid or D-Galactonic acid	9.217 ± 0.680	15.217 ± 1.815	17.174 ± 0.751	11.413 ± 1.477
Benzoic acid	5.675 ± 0.215	5.329 ± 0.288	5.027 ± 0.070	5.944 ± 0.278
**Miscellaneous**	***adg1-1/tpt-2*** **t_0 (LL)**	***adg1-1/tpt-2*** **t_{4h}**	***adg1-1/tpt-2*** **t_{48h}**	***adg1-1/tpt-2*** **(HL)**
Glycerol	1.222 ± 0.125	2.269 ± 0.164	2.330 ± 0.194	10.105 ± 3.539
myo-Inositol	7.066 ± 0.155	13.693 ± 0.513	16.346 ± 0.444	6.161 ± 0.348
Galactinol	0.127 ± 0.028	0.459 ±0.059	0.166 ± 0.010	0.073 ± 0.013
Erythritol	0.886 ± 0.019	1.572 ± 0.086	1.794 ± 0.036	1.962 ± 0.211
Tyramine	0.527 ± 0.013	0.810 ± 0.116	0.688 ± 0.016	0.588 ± 0.034
DL-Ornithine	0.712 ± 0.057	0.945 ± 0.108	1.308 ± 0.085	1.252 ± 0.116
Putrescine	1.919 ± 0.123	6.920 ± 0.981	5.177 ± 0.385	5.953 ± 0.883

Supplementary Document S1

Document S1 (Table 1). Statistical analysis (ANOVA/Tukey-Kramer) of photosynthetic electron transport (ETR) and F_v/F_m ratios of wild-type and mutant plants in a time series after transfer from LL conditions (*i.e.* a PFD of 30 $\mu mol \cdot m^{-2} \cdot s^{-1}$) to HL (*i.e.* a PFD of 300 $\mu mol \cdot m^{-2} \cdot s^{-1}$) within 172 h. The plant lines are denoted, *a* = Col-0, *b* = *tpt-2*, *c* = *adg1-1*, *d* = *adg1-1/tpt-2.* Significance levels of P < 0.05 or P < 0.01 are indicated by light or dark blue colours.

**Conditions**	**ETR**							**F_v/F_m**					
**Time**	***a vs b***	***a vs c***	***a vs d***	***b vs c***	***b vs d***	***c vs d***		***a vs b***	***a vs c***	***a vs d***	***b vs c***	***b vs d***	***c vs d***
**t_0 (LL)**													
**3h**													
**6h**													
**24h**													
**29h**													
**48h**													
**53h**													
**72h**													
**78h**													
**120h**													
**148h**													
**172h**													

Document S1 (Table 2). Statistical analysis (ANOVA/Tukey-Kramer) of starch, soluble sugar, and anthocyanin levels of wild-type and mutant plants in a time series after tansfer from LL conditions (*i.e.* a PFD of 30 µmol·m^{-2}·s^{-1}) to HL (*i.e.* a PFD of 300 µmol·m^{-2}·s^{-1}) within 148 min. The plant lines are denoted, *a* = Col-0, *b* = *tpt-2, c* = *adg1-1*, *d* = *adg1-1/tpt-2.* Significance levels of P < 0.05 or P < 0.01 are indicated by light or dark blue colours.

Condition	Starch						Sucrose						Glucose						Fructose						Condition	Anthocyanin					
Time	*a vs b*	*a vs c*	*a vs d*	*b vs c*	*b vs d*	*c vs d*	*a vs b*	*a vs c*	*a vs d*	*b vs c*	*b vs d*	*c vs d*	*a vs b*	*a vs c*	*a vs d*	*b vs c*	*b vs d*	*c vs d*	*a vs b*	*a vs c*	*a vs d*	*b vs c*	*b vs d*	*c vs d*	Time	*a vs b*	*a vs c*	*a vs d*	*b vs c*	*b vs d*	*c vs d*
t_0 (LL)		dark	dark	dark	dark																				t_0 (LL)						
30 min	dark	dark	dark	dark	dark											light				light		dark			4 h						
60 min	dark	dark	dark	dark	dark		light		light							light						dark			8 h						
120 min	dark	dark	dark	dark	dark		dark		dark	dark		dark		dark		dark		dark		dark		dark		dark	24 h	dark		dark	light		dark
240 min		dark	dark	dark	dark			light		dark		dark		dark		dark		dark		dark		dark		dark	32 h	light		dark	light		dark
480 min		dark	dark	dark	dark			dark		dark		dark		dark		dark		dark		dark		dark		dark							
HL		dark	dark	dark	dark					dark		dark		dark		dark		dark		light		dark		dark	HL		dark		dark		dark

Document S1 (Table 3). Statistical analysis (ANOVA/Tukey-Kramer) of MgProtoIX contents and Lhcb1 transcript abundance of wild-type and mutant plants in a time series after transfer from LL conditions (*i.e.* a PFD of 30 µmol·m^{-2}·s^{-1}) to HL (*i.e.* a PFD of 300 µmol·m^{-2}·s^{-1}) within 480 min. The plant lines are denoted, *a* = Col-0; *b* = *tpt-2, c* = *adg1-1*, *d* = *adg1-1/tpt-2.* Significance levels of P < 0.05 or P < 0.01 are indicated by light or dark blue colours.

**Conditions**	**MgProtoIX**							***Lhcb2***					
	a vs b	***a vs c***	***a vs d***	***b vs c***	***b vs d***	***c vs d***		***a vs b***	***a vs c***	***a vs d***	***b vs c***	***b vs d***	***c vs d***
**HL**													
**LL**													
**t_{30min}**													
**t_{60min}**													
**t_{240min}**													
**t_{480min}**													

Document S1 (Table 4). Statistical analysis (ANOVA/Tukey-Kramer) of contents of redox components of wild-type and mutant plants in a time series after transfer from LL conditions (*i.e.* a PFD of 30 µmol·m^{-2}·s^{-1}) to HL (*i.e.* a PFD of 300 µmol·m^{-2}·s^{-1}) within 4h compared to HL grown plants. The biotypes are denoted, *a* = Col-0; *b* = *tpt-2*, *c* = *adg1-1*, *d* = *adg1-1/tpt-2.* Significance levels of P < 0.05 or P < 0.01 are indicated by light or dark blue colours.

Compound	Conditions	*a vs b*	*a vs c*	*a vs d*	*b vs c*	*b vs d*	*c vs d*
**DHA**	**t_0**						
	**t_4**						
	**HL**						
**Asc**	**t_0**						
	**t_{4h}**						
	**HL**						
**DHA + ASC**	**t_0**						
	**t_4**						
	**HL**						
**GSSG**	**t_0**						
	**t_{4h}**						
	**HL**						
**GSH**	**t_0**						
	**t_{4h}**						
	**HL**						
**GSSG+GSH**	**t_0**						
	**t_{4h}**						
	**HL**						

Document S1 (Table 5). Statistical analysis (ANOVA/Tukey-Kramer) of metabolite contents of wild-type and mutant plants in a time series after transfer from LL conditions (*i.e.* a PFD of 30 µmol·m^{-2}·s^{-1}) to HL (*i.e.* a PFD of 300 µmol·m^{-2}·s^{-1}) within 4h and 48h compared to HL- or LL-grown plants. The conditions are denoted, *a* = t_0 (LL); *b* = t_{4h} (HL), *c* = t_{48h} (HL), *d* = HL. Significance levels of P < 0.05 or P < 0.01 are indicated by light or dark blue colours.

**Metabolites**	**Col-0**						***tpt-2***						***adg1-1***						***adg1-1/tpt-2***					
**Sugars**	*a vs b*	*a vs c*	*a vs d*	*b vs c*	*b vs d*	*c vs d*	*a vs b*	*a vs c*	*a vs d*	*b vs c*	*b vs d*	*c vs d*	*a vs b*	*a vs c*	*a vs d*	*b vs c*	*b vs d*	*c vs d*	*a vs b*	*a vs c*	*a vs d*	*b vs c*	*b vs d*	*c vs d*
Sucrose, D- (8TMS)	dark blue	dark blue	dark blue	dark blue	dark blue	light blue	light blue	dark blue	dark blue	dark blue	dark blue	dark blue	dark blue	dark blue	dark blue		dark blue	dark blue	dark blue	dark blue	dark blue	light blue		dark blue
Glucose, D- (1MEOX) (5TMS)	dark blue	dark blue	dark blue	dark blue	dark blue		light blue	dark blue	dark blue	dark blue	dark blue	dark blue	dark blue	dark blue	dark blue			dark blue	dark blue	dark blue			dark blue	dark blue
Fructose, D- (1MEOX) (5TMS)	dark blue	dark blue	dark blue	dark blue			dark blue	dark blue	dark blue	dark blue	dark blue	dark blue	dark blue	dark blue	dark blue				light blue	dark blue		dark blue	light blue	dark blue
Mannose, D- (1MEOX) (5TMS)							dark blue	dark blue	dark blue	dark blue		light blue	light blue	dark blue	dark blue	dark blue	dark blue	dark blue	dark blue	dark blue			dark blue	light blue
Maltose, D- (1MEOX) (8TMS)		dark blue		dark blue		dark blue		dark blue	dark blue	dark blue	dark blue	dark blue	dark blue	dark blue	dark blue		dark blue	dark blue	dark blue	dark blue	light blue	dark blue	dark blue	
Trehalose, alpha,alpha'-, D- (8TMS)		dark blue	dark blue	dark blue	dark blue	dark blue		dark blue	dark blue	dark blue	dark blue	light blue		dark blue	dark blue	dark blue	dark blue	dark blue			dark blue		dark blue	dark blue
Raffinose (11TMS)			dark blue		dark blue	dark blue	dark blue	light blue			dark blue		dark blue	dark blue	dark blue	dark blue	light blue		dark blue			dark blue	dark blue	
Glucose, 1,6-anhydro, beta-D- (3TMS)		dark blue		light blue			light blue	dark blue	dark blue				light blue	dark blue	dark blue				dark blue	dark blue			dark blue	dark blue
Mannopyranoside, 1-O-methyl-, alpha-D- (4TMS)		dark blue	dark blue	dark blue	dark blue	dark blue		dark blue	dark blue	light blue				dark blue	dark blue	dark blue	dark blue	dark blue		dark blue		light blue		light blue
Fucose, DL- (1MEOX) (4TMS)	dark blue	dark blue	dark blue	dark blue	dark blue		light blue	dark blue	dark blue	dark blue		dark blue	dark blue	dark blue	dark blue	dark blue	dark blue		dark blue			light blue	dark blue	
Arabinose, D- (1MEOX) (4TMS)	dark blue	light blue	dark blue	dark blue	dark blue				dark blue		dark blue	dark blue								dark blue		dark blue	light blue	light blue
Glucoheptose (1MEOX) (6TMS)	dark blue	dark blue	dark blue	dark blue			dark blue	dark blue					dark blue	dark blue	dark blue	dark blue	dark blue	dark blue	dark blue	dark blue			dark blue	dark blue

Document S1 (Table 5), continued

Amino acids

Pyroglutamic acid / Glutamic acid																								
Aspartic acid, L- (3TMS)																								
Asparagine, DL- (3TMS)																								
Alanine, DL- (3TMS)																								
Glycine (3TMS)																								
Serine, DL- (3TMS)																								
Threonine, DL- (3TMS)																								
Cysteine, DL- (3TMS)																								
Methionine, DL- (2TMS)																								
Isoleucine, L- (2TMS)																								
Valine, DL- (2TMS)																								
Lysine, L- (4TMS)																								
Arginine, DL-, -NH3 (3TMS)																								
Phenylalanine, DL- (2TMS)																								
Tyrosine, DL- (3TMS)																								
Tryptophan, L- (2TMS)																								
Proline, L- (2TMS)																								
Alanine, beta- (3TMS)																								

Document S1 (Table 5), continued

**Organic acids**																								
Pyruvic acid (1MEOX) (1TMS)																								
Malic acid, 2-methyl-, DL- (3TMS)																								
Glutaric acid (2TMS)																								
Succinic acid (2TMS)																								
Fumaric acid (2TMS)																								
Malonic acid (2TMS)																								
Glyceric acid, DL- (3TMS)																								
Butyric acid, 3-hydroxy- (2TMS)																								
Gluconic/Galactonic acid (6TMS)																								
Benzoic acid (1TMS)																								

Document S1 (Table 5), continued.

Others
Glycerol (3TMS)
Inositol, myo- (6TMS)
Galactinol (9TMS)
Erythritol (4TMS)
Tyramine (3TMS)
Ornithine, DL- (4TMS)
Putrescine (4TMS)

Document S1 (Table 6). Statistical analysis (ANOVA/Tukey-Kramer) of metabolite contents of wild-type and mutant plants in a time series after transfer from LL conditions (*i.e.* a PFD of 30 µmol·m^{-2}·s^{-1}) to HL (*i.e.* a PFD of 300 µmol·m^{-2}·s^{-1}) within 4h and 48h compared to HL- or LL-grown plants. The plant lines are denoted, *a* = Col-0; *b* = *tpt-2*, *c* = *adg1-1*, *d* = *adg1-1/tpt-2.* Significance levels of P < 0.05 or P < 0.01 are indicated by light or dark blue colours.

**Metabolites**	**t_0 (LL)**						**t_{4h} (HL)**						**t_{48h} (HL)**						**HL**					
**Sugars**	***a vs b***	***a vs c***	***a vs d***	***b vs c***	***b vs d***	***c vs d***	***a vs b***	***a vs c***	***a vs d***	***b vs c***	***b vs d***	***c vs d***	***a vs b***	***a vs c***	***a vs d***	***b vs c***	***b vs d***	***c vs d***	***a vs b***	***a vs c***	***a vs d***	***b vs c***	***b vs d***	***c vs d***
Sucrose, D- (8TMS)																								
Glucose, D- (1MEOX) (5TMS)																								
Fructose, D- (1MEOX) (5TMS)																								
Mannose, D- (1MEOX) (5TMS)																								
Maltose, D- (1MEOX) (8TMS)																								
Trehalose, alpha,alpha'-, D- (8TMS)																								
Raffinose (11TMS)																								
Glucose, 1,6-anhydro, beta-D- (3TMS)																								
Mannopyranoside, 1-O-methyl-, alpha-D- (4TMS)																								
Fucose, DL- (1MEOX) (4TMS)																								
Arabinose, D- (1MEOX) (4TMS)																								
Glucoheptose (1MEOX) (6TMS)																								

Document S1 (Table 6), continued

Amino acids
Pyroglutamic acid / Glutamic acid
Aspartic acid, L- (3TMS)
Asparagine, DL- (3TMS)
Alanine, DL- (3TMS)
Glycine (3TMS)
Serine, DL- (3TMS)
Threonine, DL- (3TMS)
Cysteine, DL- (3TMS)
Methionine, DL- (2TMS)
Isoleucine, L- (2TMS)
Valine, DL- (2TMS)
Lysine, L- (4TMS)
Arginine, DL-, -NH3 (3TMS)
Phenylalanine, DL- (2TMS)
Tyrosine, DL- (3TMS)
Tryptophan, L- (2TMS)
Proline, L- (2TMS)
Alanine, beta- (3TMS)

Document S1 (Table 6), continued

**Organic acids**																								
Pyruvic acid (1MEOX) (1TMS)																								
Malic acid, 2-methyl-, DL- (3TMS)																								
Glutaric acid (2TMS)																								
Succinic acid (2TMS)																								
Fumaric acid (2TMS)																								
Malonic acid (2TMS)																								
Glyceric acid, DL- (3TMS)																								
Butyric acid, 3-hydroxy- (2TMS)																								
Gluconic/Galactonic acid (6TMS)																								
Benzoic acid (1TMS)																								

Document S1 (Table 6), continued

**Others**																								
Glycerol (3TMS)																								
Inositol, myo- (6TMS)																								
Galactinol (9TMS)																								
Erythritol (4TMS)																								
Tyramine (3TMS)																								
Ornithine, DL- (4TMS)																								
Putrescine (4TMS)																								

Supplementary Document S2

Static assessment of global gene expression after LL/HL-transfer

(A) *adg1-1 vs* Col-0

The starch-free *adg1-1* single mutant contained the highest number of significantly altered genes in the static comparison, particularly under LL-conditions (Fig. 6A) or 48h after LL/HL-transfer. Under LL-conditions 16 gens belonging to the category \`protein metabolism´ were differentially regulated as a part of the 144 highly altered genes. Interestingly, among these 16 genes, 12 genes related to \`protein degradation´ were down-regulated and four genes related to \`protein synthesis´ were up-regulated (Tables 1, Supplementary Table S2A), suggesting that in the absence of starch the maintenance of protein abundance is promoted.

The transfer from LL to HL had a profound impact on the functional categories of altered genes in the *adg1-1* single mutant. Although the number of differentially regulated genes dropped 4h after transfer to HL, 30 and 17 genes were up- and down-regulated, respectively, compared to the wild type (Table 1, Supplementary Table S3A). Among the 30 up-regulated genes, there were five genes related to \`lipid metabolism´. Four of the five proteins reside in chloroplasts. Furthermore, four genes involved in \`secondary metabolism´ (flavonoid biosynthesis) were up-regulated and five genes related to \`stress´ were down-regulated (Table 1, Supplementary Table S3A). These data suggest an elevated input into lipid formation and/or secondary metabolism as a consequence of a deficiency in the night-path of photoassimilate export from the chloroplast.

The functional pattern of genes changed appreciably after 48h in HL (Fig. 6, Table 1, Supplementary Table S4A). There were a total of 209 genes specifically altered in *adg1-1* compared to the wild type. Interestingly, although the category \`lipid metabolism´ was missing among the 58 highly up-regulated genes, there were seven genes belonging to this category among the 151 highly down-regulated genes, with four of these genes related to \`lipid degradation´. Hence, the transient enhancement of lipid synthesis (i.e. 4h after LL/HL-transfer) was replaced by an inhibition of lipid degradation in the long term. Furthermore five genes involved in \`major CHO metabolism´, 12 genes related to \`proteins´ (including seven genes connected to \`protein synthesis´), as well as six genes related to \`regulation of transcription (RT)´ were highly up-regulated (Table 1). Moreover, the up-regulation of genes involved in \`protein synthesis´ was accompanied by a down-regulation of 13 genes related to \`protein degradation´, again suggesting an enhanced production and/or maintenance of proteins. Furthermore 21, 11, and seven genes related to \`RT´, \`stress´ and \`signalling´,

respectively, were highly down-regulated in *adg1-1* 48h after LL/HL-transfer (Supplementary Table S4A). Moreover, the gene coding for the glucose 6-phosphate/phosphate translocator 2 (*GPT2*; At1g61800) was highly up-regulated in *adg1-1*.

(B) *tpt-2 vs* Col-0

Under LL-conditions, a limitation in the day-path of photoassimilate export from the chloroplast in the *tpt-2* mutant resulted only in the down-regulation of a single gene encoding a disulfide isomerase-like protein (AtPDIL 5-4, At4g27080; Supplementary Table S2B).

The number of highly altered genes in *tpt-2* was increased to 36, 4h after the plants were transferred to HL. There were only two genes down-regulated, amongst them again AtPDIL 5-4 and a protein of unknown function (At4g27080; Supplementary Table S3B). The 34 highly up-regulated genes comprised three genes related to \`major carbohydrate metabolism´ and seven, significantly over-represented genes involved in \`protein degradation´ (Table 1). After 48h in HL, again, there were more genes highly up-regulated (104) than down-regulated (3) (Supplementary Table S4B). Interestingly, AtPDIL 5-4 still belonged to the down-regulated genes. Within the group of up-regulated genes there were three significantly over-represented functional clusters, *i.e.* \`cell wall´, \`hormone metabolism´ and \`stress´ (Table 1). Furthermore, genes related to \`RT´ and \`development´ were represented with at least five members. Ten genes, including a MAPkinase (At1g01560), were connected to \`signalling´, in particular, \`calcium signalling´ (nine genes).

(c) *adg1-1/tpt-2 vs* Col-0

Despite the relative high number of specifically altered genes in the *adg1-1* mutant, surprisingly, the combined deficiency in the day- and night-path of photoassimilate export resulted in an appreciable lower number of differentially regulated genes in *adg1-1/tpt-2*. Under LL-conditions there were only 21 genes highly up- or down-regulated in the double mutant (Supplementary Table S2C). Among the nine up-regulated genes in *adg1-1/tpt-2,* remarkably, there were three genes related to \`abiotic stress´, sub-category heat. All three genes belong to the putative HSP20-type protein of unknown function (At1g53540, At3g46230, At5g12020). Of the 12 down-regulated genes one half is related to \`histone proteins´ and \`chromatin structure´, suggesting that parts of the DNA was not associated with proteins and/or the plants contained less DNA. The only gene dramatically down-regulated in the overlapping area of *adg1-1/tpt-2* and *tpt-2* was, as expected, the *TPT* gene (Supplementary Table S2D). In the overlapping region between *adg1-1/tpt-2* and *adg1-1*

there were 13 up- and 33 down-regulated genes found. Among the down-regulated genes there were six genes related to \`chromatin structure´ as well as five genes involved in \`protein degradation´, again suggesting a function of starch and/or soluble sugars in protein maintenance and chromatin structure.

After 4h in HL only 10 genes were highly altered in *adg1-1/tpt-2* (Fig. 6B, Supplementary Table S3C). The three up-regulated genes comprised a stress induced protein (At2g40170) involved in ABA metabolism and a chloroplast localised superoxide dismutase (At2g28190). Both genes were more pronounced up-regulated in *adg1-1/tpt-2* compared to *adg1-1* or *tpt-2*. Amongst the down-regulated genes there was a bHLH-type transcription factor (At4g17880; MYC4), which was highly and specifically down-regulated with a log2-ratio of -5.17 in *adg1-1/tpt-2.* Strikingly the same gene was also highly down-regulated after 48h in HL (log2-ratio = -4.21) and even in LL (log2-ratio = -3.68). A closer inspection of the expression profiles (eFP browser, Winter *et al.*, 2007) revealed that this gene is highly regulated by various stress conditions, like for instance oxidative stress, and it responds to jasmonate (Fernández-Calvo *et al*., 2011). Moreover, the presence of externally fed Suc induces the expression of At4g17880. Furthermore, MYC4 has been identified to be one of the key players in the regulation of glucosinolate biosynthesis (Schweizer *et al.,* 2013). At t_{48h} there were eight more transcriptional regulators within the group of 112 highly down-regulated genes in *adg1-1/tpt-2*. Moreover, the functional categories \`amino acids´, \`cell wall´, and \`major carbohydrate metabolism´ were significantly over-represented (Table 1). Interestingly, among the 36 highly up-regulated genes, 22 were plastome-encoded and belonged to the categories \`PS light reaction´ (16 genes), \`protein biosynthesis´ (five genes) and \`lipid metabolism´ (one gene). Moreover, there were four nuclear-encoded genes involved in \`RT´ up-regulated in *adg1-1/tpt-2*.

Supplementary Document S3

Genes associated with `major carbohydrate´- and `lipid metabolism´ as well as `transport´ were commonly up-regulated 4h after LL/HL-transfer

The group of commonly regulated genes as a response to LL/HL-transfer comprised also metabolic genes. Strikingly, seven and 11 genes associated with `lipid-´ and `major CHO metabolism´, respectively, were up-regulated only transiently at t_{4h} *vs* t_0 (Supplementary Table S7A).

Among the up-regulated genes associated with `lipid metabolism´, there were two genes involved in triacylglycerol (TAG) biosynthesis (At1g54570 and At2g19450), a sterol oxidase (At1g07420), probably anchored at the outer envelope (gene ontology, cellular component), a 16:0 delta9 desaturase (At2g31360) and a phospholipase A2 family protein (At2g06925). As an analysis with ATTED-II (version 6.1) revealed, all genes apart from a plastidial thioesterase (At1g54570) belong to a regulatory network (Supplementary Fig. S3B; Supplementary Table S7A).

Moreover, in the category `lipid metabolism´ there were ten and seven genes down-regulated at t_{4h} *vs* t_0 and t_{48h} *vs* t_0, respectively, with an overlap of five genes. The ten down-regulated genes at t_{4h} *vs* t_0 form, with the exception of At5g08030, a large network (Supplementary Fig. S3C), whereas there is no evidence for any exceptional network formation with the seven down-regulated genes at t_{48h} *vs* t_0 (not shown).

In the category `major CHO metabolism´, the 11 up-regulated genes at t_{4h} *vs* t_0 were - in a broader sense - all involved in either starch synthesis or degradation, despite the fact that the *adg1-1* single mutant and the *adg1-1/tpt-2* double mutant lack starch (Supplementary Table S7A). Furthermore, all 11 genes belong to a single regulatory network (Supplementary Fig. S2A). The list of highly up-regulated genes comprised not only chloroplast-localised metabolic enzymes such as α-amylase (At1g69830), disproportionating enzyme 1 (DPE1, At5g64860; Stettler *et al.,* 2009), isoamylase/debranching enzyme (At4g09020; Streb *et al.,* 2008; Wattebled *et al.,* 2008), branching enzyme 1 and 2 (At3g20440; At2g36390; Walters *et al.,* 2004; Dumez *et al.,* 2006) and glucan phosphorylase (At3g29320), but also regulatory proteins such as glucan water dikinase (GWD; SEX1; At1g10760; Yu *et al.,* 2001) phosphoglucan, water dikinases (PWD; AtGWD3; At5g26570; Kötting *et al.,* 2005), involved in the phosphorylation of glucose residues in amylopectin at C_6 and C_3 (Ritte *et al.,* 2006), respectively, and a protein phosphatase (AtSEX4; At3g52180), involved in the dephosphorylation of the aforementioned glucose residues (Hejazi *et al.,* 2010). Moreover,

two genes associated with the cytosolic conversion of maltose to sucrose (*i.e.* disproportionating enzyme 2 [At2g40840; Lu & Sharkey, 2004] and glucan posphorylase 2 [At3g46970]) were highly up-regulated 4h after LL/HL-transfer in both wild-type and mutant plants. Again this regulation of genes involved in carbohydrate metabolism also occurred in the starch-free background (*i.e. adg1-1* and *adg1-1/tpt-2*). Interestingly, three of the starch-related genes belong to the co-expression network of phospholipase A (At2g06925; Supplementary Table S7A). Moreover, within the co-regulation network of genes belonging to the category \`major CHO metabolism´, there was a chloroplast localised AMP activated protein kinase induced 4h and 48h after transfer to HL (Supplementary Table S7, A and B). The respective gene (At5g39790) appears to contain a starch binding domain (SUBA3 database; Heazlewood *et al.,* 2007; Tanz *et al*., 2013) and might hence be involved in carbohydrate metabolism or signalling. Most strikingly, the expression of \`starch related´ genes occurred independently from the presence of starch (*i.e.* in the starch-free background *adg1-1*), suggesting that the resulting proteins might have additional unknown functions.

In the category \`secondary metabolism´ there were five and 11 genes up-regulated, related to \`flavonoids´ in a broader sense at t_{4h} *vs* t_0 and t_{48h} *vs* t_0, respectively (Supplementary Table S7, A and B). Only three of these genes were commonly differently regulated at both time points. In addition, three more up-regulated genes belonged to the sub-category \`isoprenoids´ and \`miscellaneous´.

Genes associated with specific \`transport processes´ were differentially expressed most pronounced 4h after LL/HL-transfer

Genes associated with \`transport processes´ were de-regulated both at 4h and 48h after LL/HL-transfer. Of the 14 up-regulated genes at t_{4h} *vs* t_0, only two genes were also found at t_{48h} *vs* t_0. Likewise, of the 18 down-regulated transport associated genes at t_{4h} *vs* t_0, only six were also found at t_{48h} *vs* t_0 (Supplementary Table S7, A and B). Interestingly two genes belonging to the phosphate translocator family were de-regulated after 4h in HL. The glucose 6-phosphate/phosphate translocator2 (GPT2; At1g61800) and the phosphoenolpyruvate/phosphate translocator2 (PPT2; At3g01550) were up- and down-regulated, respectively. It has been demonstrated that *GPT2*, which is usually only expressed in generative tissue (eFP browser; Winter *et al.,* 2007), strongly responds to elevated soluble sugar levels (Kunz *et al.,* 2010, Schmitz *et al.,* 2012), *e.g.* in starch-free mutants or after feeding of exogenous sugars to the plants (Heinrichs *et al.,* 2012). *GPT2* was highly up-regulated with log2-ratios between 3.7 and 6.7 at t_{4h} *vs* t_0 in all plant lines

(Supplementary Table S7). At 48h after LL-to-HL-transfer, the log2-ratios of *GPT2* expression in the wild-type and the *tpt-2* single mutant dropped to lower levels compared to t_{4h} *vs* t_0. However, in the *adg1-1* mutant the expression of *GPT2* was further increased from 6.7 at t_{4h} *vs* t_0 to 7.2 at t_{48h} *vs* t_0, whereas in the double mutant *GPT2* was not significantly altered. The changes in the *GPT2* expression ratios in the time series correspond well with levels of soluble sugars in wild-type and mutant plants (Compare Fig. 1 and Table 4).

Of the 14 up-regulated transport-related genes, six genes encode proteins with a high probability of a mitochondrial localisation and only two are likely to be localised in the chloroplasts. The portion of organelle-localised gene products was further diminished in the group of down-regulated transport related genes (*i.e.* two mitochondrial and one plastidial).

Additional References (Supplementary Document S3)

Dumez S, Wattebled F, Dauvillee D, Delvalle D, Planchot V, Ball SG, D'Hulst C. 2006. Mutants of Arabidopsis lacking starch branching enzyme II substitute plastidial starch synthesis by cytoplasmic maltose accumulation. *The Plant Cell* **18,** 2694-2709. http://www.ncbi.nlm.nih.gov/pubmed/17028209

Heazlewood JL, Verboom RE, Tonti-Filippini J, Small I and Millar AH. 2007. SUBA: the Arabidopsis Subcellular Database. *Nucleic Acids Research.* **35(D),** 213-218. http://www.ncbi.nlm.nih.gov/pubmed/17071959

Hejazi M, Fettke J, Kötting O, Zeeman SC, Steup M. 2010. The Laforin-like dual-specificity phosphatase SEX4 from Arabidopsis hydrolyzes both C6- and C3-phosphate esters introduced by starch-related dikinases and thereby affects phase transition of alpha-glucans. *Plant Physiology* **152,** 711-722. http://www.ncbi.nlm.nih.gov/pubmed/20018599

Kötting O, Pusch K, Tiessen A, Geigenberger P, Steup M, Ritte G. 2005. Identification of a novel enzyme required for starch metabolism in Arabidopsis leaves. The phosphoglucan, water dikinase. Plant Physiology **137,** 242-252. http://www.ncbi.nlm.nih.gov/pubmed/15618411

Lu Y, Sharkey TD. 2004. The role of amylomaltase in maltose metabolism in the cytosol of photosynthetic cells. *Planta* **218,** 466–473 http://www.ncbi.nlm.nih.gov/pubmed/14593480

Ritte G, Heydenreich M, Mahlow S, Haebel S, Kötting O, Steup M. 2006. Phosphorylation of C6- and C3-positions of glucosyl residues in starch is catalysed by distinct dikinases. FEBS Letters **580,** 4872-4876. http://www.ncbi.nlm.nih.gov/pubmed/16914145

Stettler M, Eicke S, Mettler T, Messerli G, Hortensteiner S, Zeeman SC. 2009. Blocking the metabolism of starch breakdown products in Arabidopsis leaves triggers chloroplast degradation. *Molecular Plant* **2,** 1233-1246. http://www.ncbi.nlm.nih.gov/pubmed/19946617

Streb S, Delatte T, Umhang M, Eicke S, Schorderet M, Reinhardt D, Zeeman SC. 2008. Starch granule biosynthesis in Arabidopsis is abolished by removal of all debranching enzymes but restored by the subsequent removal of an endoamylase. *The Plant Cell.* **20,** 3448-3466. http://www.ncbi.nlm.nih.gov/pubmed/19074683

Tanz SK, Castleden I, Hooper CM, Vacher M, Small I; Millar, AH. 2013. SUBA3: a database for integrating experimentation and prediction to define the SUBcellular location of proteins in Arabidopsis. *Nucleic Acids Research.* **41(D),** 1185-1191. http://www.ncbi.nlm.nih.gov/pubmed/23180787

Wattebled F, Planchot V, Dong Y, Szydlowski N, Pontoire B, Devin A, Ball S, D'Hulst C. 2008. Further evidence for the mandatory nature of polysaccharide debranching for the aggregation of semicrystalline starch and for overlapping functions of debranching enzymes in Arabidopsis leaves. *Plant Physiology* **148,** 1309-1323. http://www.ncbi.nlm.nih.gov/pubmed/18815382

Yu T-S, Kofler H, Häusler RE, Hille D, Flügge UI, Zeeman SC, Smith AM, Kossmann J, Lloyd J, Ritte G, Steup M, Lue W-L, Weber A. 2001. The Arabidopsis *sex1* mutant is defective in the R1 protein, a general regulator of starch degradation in plants, and not the chloroplast hexose transporter. *The Plant Cell* **13,** 1907-1918. http://www.ncbi.nlm.nih.gov/pubmed/11487701
